# Single-cell RNA sequencing reveals regulatory mechanism for trophoblast cell-fate divergence in human peri-implantation conceptuses

**DOI:** 10.1371/journal.pbio.3000187

**Published:** 2019-10-09

**Authors:** Bo Lv, Qin An, Qiao Zeng, Xunyi Zhang, Ping Lu, Yanqiu Wang, Xianmin Zhu, Yazhong Ji, Guoping Fan, Zhigang Xue

**Affiliations:** 1 Department of Regenerative Medicine, School of Medicine, Tongji University, Shanghai, China; 2 Department of Human Genetics, David Geffen School of Medicine, University of California Los Angeles, Los Angeles, State of California, United States of America; 3 Center of Reproductive Medicine of Ji’an Maternal and Child Health Hospital, Ji’an, Jiangxi, China; 4 Reproductive Medicine Center, Tongji Hospital, Tongji University, Shanghai, China; 5 Shanghai Pulmonary Hospital, School of Life Sciences and Technology, Tongji University, Shanghai, China; California Institute of Technology, UNITED STATES

## Abstract

Multipotent trophoblasts undergo dynamic morphological movement and cellular differentiation after conceptus implantation to generate placenta. However, the mechanism controlling trophoblast development and differentiation during peri-implantation development in human remains elusive. In this study, we modeled human conceptus peri-implantation development from blastocyst to early postimplantation stages by using an in vitro coculture system and profiled the transcriptome of 476 individual trophoblast cells from these conceptuses. We revealed the genetic networks regulating peri-implantation trophoblast development. While determining when trophoblast differentiation happens, our bioinformatic analysis identified T-box transcription factor 3 (TBX3) as a key regulator for the differentiation of cytotrophoblast (CT) into syncytiotrophoblast (ST). The function of TBX3 in trophoblast differentiation is then validated by a loss-of-function experiment. In conclusion, our results provided a valuable resource to study the regulation of trophoblasts development and differentiation during human peri-implantation development.

## Abstract 2

After implantation of the conceptus, trophoblasts undergo dynamic movements and cellular differentiation to generate the placenta, but the mechanisms controlling this process in humans remain elusive. In this study, single-cell RNA-seq of human peri-implantation conceptuses in vitro reveals TBX3 as a key regulator of trophoblast cell-fate divergence.

## Introduction

The placenta is the interface between the fetal and maternal circulation and plays an essential role in supporting fetus development and survival. Most placental functions are carried out by trophoblasts, which are derived from the trophectoderm (TE) in blastocysts [1]. Placenta development in the human is a multistep process involving dynamic morphological movement and cellular differentiation of trophoblasts. In the human, blastocysts first adhere to the endometrium by the polar TE, which is the embryonic pole adjacent to inner cell mass [2]. After adhesion, the multipotent trophoblast cells at the polar TE migrate through the endometrial (EM) cells and differentiate into initial multinucleated syncytiotrophoblast (ST), where small vacuoles then develop into the lacunar system and finally become the intervillous space. The remaining of trophoblast cells stay multipotent and are called cytotrophoblast (CT) [2]. On about the 12th day post fertilization, cytotrophoblastic proliferation increases and CT cells invade the ST to form the primary anchoring villi and free villi. Mesenchymal cells then begin to invade the primary villi, transforming them into the secondary villi. After that at about 20th day post fertilization, the fetal capillaries then appear, which marks the development of the tertiary villi. Along with all these steps, inner CT cells continue to differentiate and fuse to form the outer ST cells, and the invasive extravillous trophoblast (EVT) cells form at the tips of the anchoring villi. Until term, all the vascularized villi can be categorized as the tertiary villi [3].

Previous anatomical studies using a relatively small number of human specimens have shown that the CT appears right after implantation, which then differentiates into the EVT and ST before day 21 postfertilization [2, 3]. Recent studies using single-cell RNA sequencing (RNA-seq) on the human placenta successfully revealed transcriptomic and functional heterogeneity between different trophoblast sublineages within the placenta. However, all these single-cell profiling studies used placenta after 6 gestational weeks [4–7]. Because of the lack of data for early postimplantation conceptuses, it is still elusive when trophoblast sublineages are established and how the trophoblast differentiation is regulated in the human.

Human conceptuses cocultured with EM cells in vitro can model morphological and molecular changes of the peri-implantation conceptuses in vivo [8–10]. Although in vitro cultured embryos show limited development of epiblast (EPI) cells, they can well recapitulate the TE-lineage differentiation when coculturing with EM cells [10–13]. In this study, we profiled transcriptomes of 476 individual trophoblast cells isolated from 19 human conceptuses cocultured with EM cells. We revealed the regulatory networks underlying trophoblast development and differentiation. Using a unique bioinformatic approach and loss-of-function verification experiments, we identified a novel transcription factor T-box transcription factor 3 (TBX3) that is required by ST development. Our results provided a rich resource to study the early placenta development.

## Results

### Single-cell transcriptome profiling of trophoblasts in human peri-implantation conceptuses

**Fig 1:**
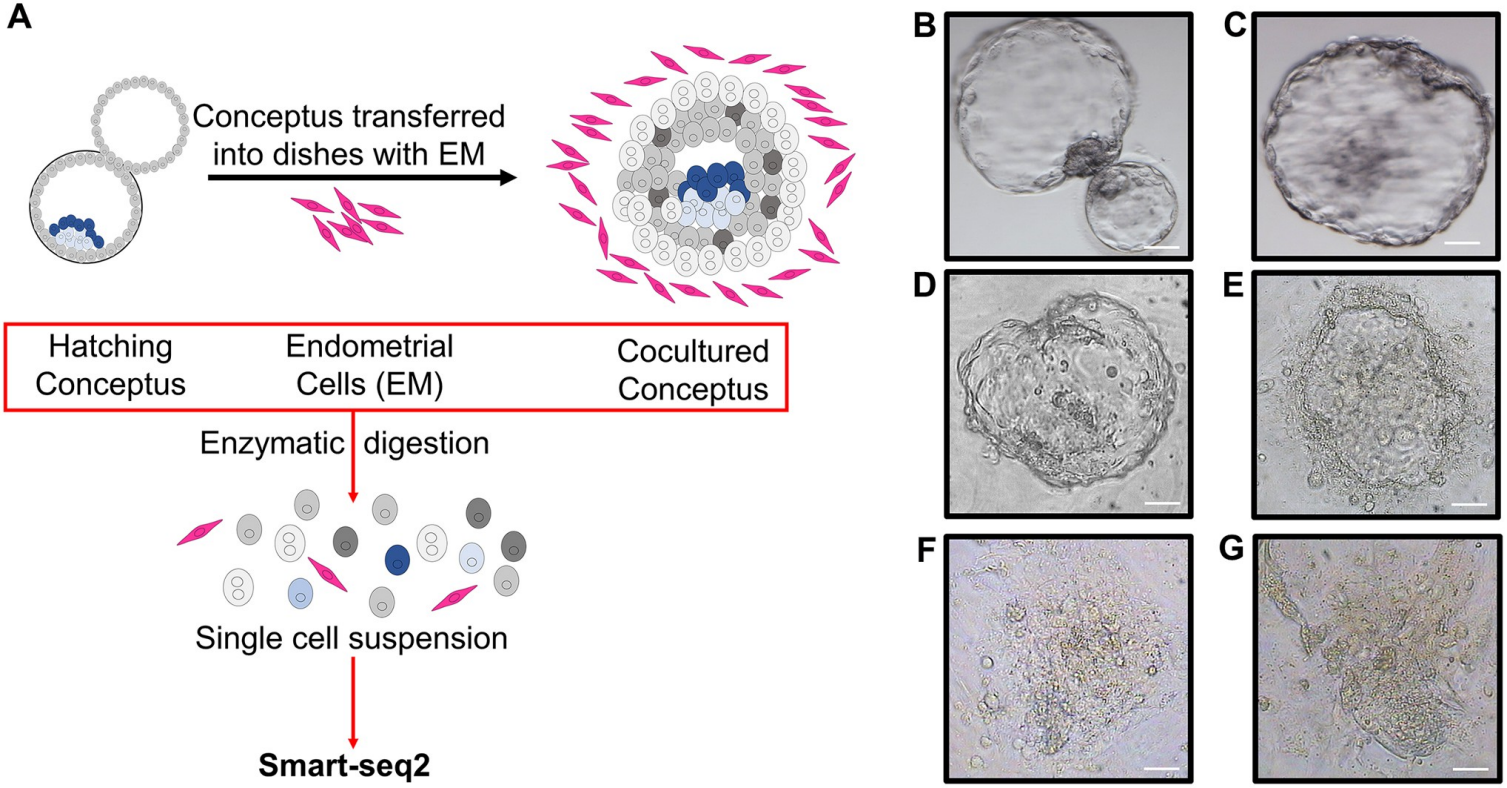
Experiment design of the study. (A) Human blastocysts generated using in vitro fertilization were cocultured with human primary EM cells. EM cells and conceptuses were dissociated and individual cells were collected for single-cell RNA sequencing using SMART-seq2 protocol. (B–G) Representative microscopic images of human peri-implantation conceptuses, including day 6 conceptuses (B), day 7 un-cocultured (C) and cocultured (D) conceptuses, day 8 conceptuses (E), day 9 conceptuses (F), and day 10 conceptuses (G). (Scale bars = 100μm.) EM, endometrial.

We first obtained human peri-implantation conceptuses by coculturing blastocysts with human primary EM cells (Fig 1A). Briefly, conceptuses generated by in vitro fertilization were first cultured to the blastocyst stage following the method described before by Shahbazi and colleagues and Deglincerti and colleagues [11, 12]. At the blastocyst stage (day 6.5), conceptuses were transferred to culture dishes plated with human primary EM cells. At day 8, all cocultured conceptuses hatched out from zona pellucida, attached to the bottom of the dish, and adopted a flattened structure that is very similar to previous reports [11, 12] (Fig 1B and 1G).

**Fig 2:**
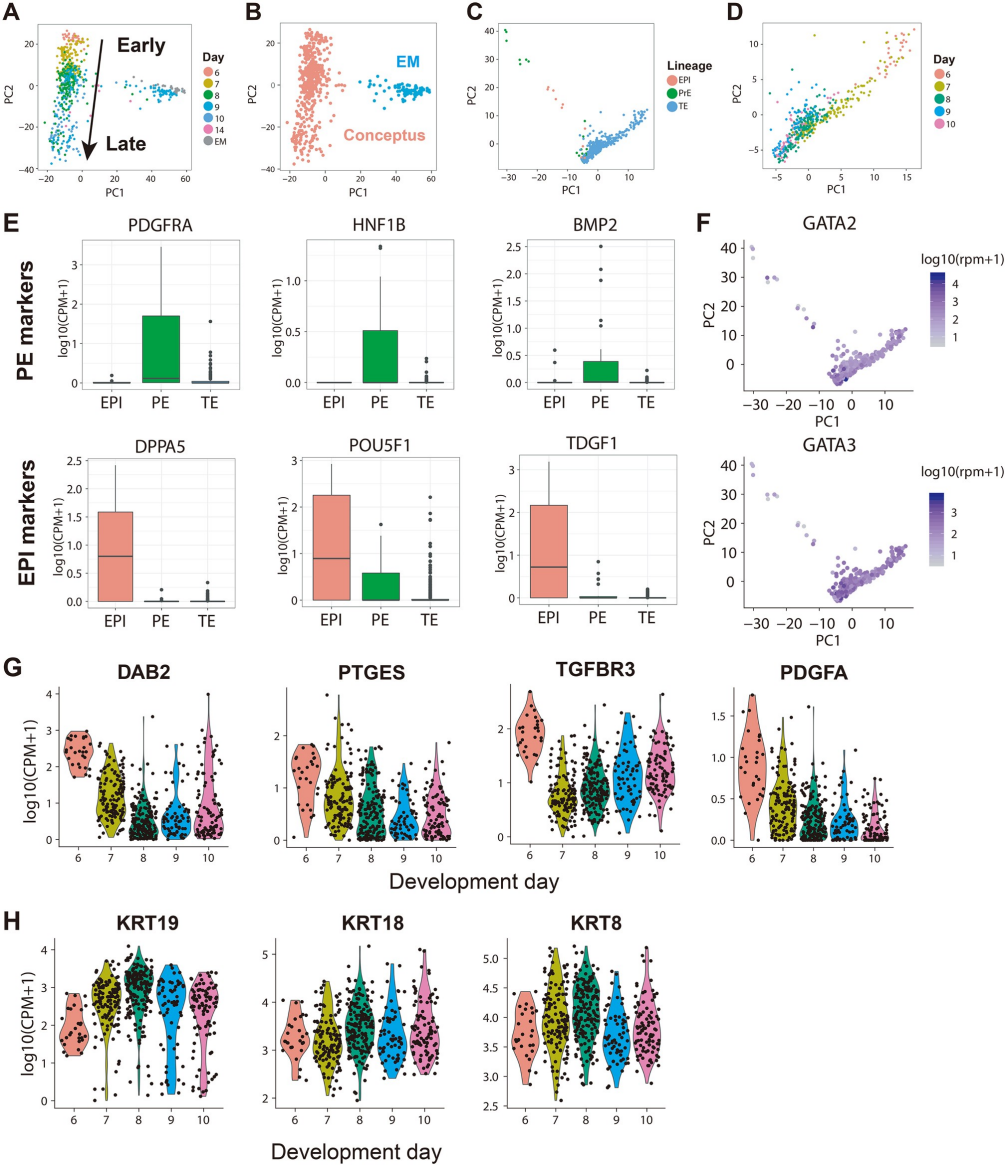
Single-cell RNA sequencing revealed maintained marker genes for trophoblasts during peri-implantation development. (A) PCA showed that cells form 2 distinct clusters, representing conceptus cells and EM cells. (B) Some EM cells were annotated as conceptus cells during manual cell picking. Hierarchical clustering using transcriptome data unbiasedly separated conceptus cells and EM cells, and the new cell identities were visualized on PCA. (C) Conceptus cells were classified into EPI-, PE-, or TE-lineage cells based on their expression of 300 previously defined lineage markers. The cell lineage identities were visualized on PCA computed using the 300 lineage marker genes. (D) PCA showing the TE cells across development days using 300 genes. (E) Box plot showing the expression of representative lineage markers in EPI- or PE-lineage cells we defined. (F) Scatter plot related to Fig 2C using the 300 lineage marker genes showing the 2 classic TE marker genes, namely, *GATA2* and *GATA3*, were sustainably expressed in TE and trophoblasts during peri-implantation development. (G) Violin plot showing the expression of 4 previously reported TE-lineage markers during peri-implantation development. (H) Violin plot showing the expression of 3 highly expressed TE-lineage markers we identified during peri-implantation development. EM, endometrial; EPI, epiblast; PCA, principle component analysis; PE, primitive endoderm; TE, trophectoderm.

To obtain transcriptomic profiles of human trophoblast cells during peri-implantation development, we harvested single cells from 19 conceptuses from day 6 to day 10, complemented with 25 EM cells. Transcriptomes from 614 single cells were successfully profiled, with 0.7 million uniquely mapped reads and 24,011 detected transcripts per cell on average. Principle component analysis (PCA) and unbiased hierarchical clustering showed that conceptus cells and EM cells form 2 distinct clusters (Fig 2A), and some EM cells were mislabeled as conceptus cells during cell picking (Fig 2B). Therefore, we excluded all EM cells and focused on 516 conceptus cells in our subsequent analysis.

The 516 conceptus cells consist of cells of EPI, primitive endoderm (PE) and TE lineages. To separate trophoblasts that derived from TE lineage from EPI- and PE-lineage cells, we determined lineage origination of each cell based on its expression of 300 lineage markers, using an algorithm reported before by Petropoulos and colleagues [14]. We identified 476 TE-, 14 EPI- and 26 PE-lineage cells. PCA analysis using the 300 lineage markers showed that EPI and PE cells separated from TE-lineage cells (Fig 2C and 2D, S1A and S1C Fig, and S1 Table). In addition, EPI and PE cells have higher expression of their corresponding maker genes compared with TE (Fig 2E), whereas well-characterized TE markers such as *GATA2* and *GATA3* were highly expressed in all TE-lineage cells (Fig 2F). These results suggested that this algorithm faithfully separated EPI and PE-lineage cells from TE-lineage cells.

Interestingly, whereas *GATA2* and *GATA3* were highly expressed through day 6 to day 10, the expression of other TE markers such as *DAB2*, *PTGES*, *TGFBR3*, and *PDGFA* were significantly down-regulated after implantation at day 7 (Fig 2G, S1D Fig, and S2 Table). These results have multiple possible interpretations: it may because not all TE-lineage markers are sustainably expressed in trophoblasts during peri-implantation development, or only a restricted number of trophoblast populations were expanded, or that these genes are aberrantly shut down in in vitro cultured human conceptuses. We found 5 TE markers, namely, *KRT19*, *KRT18*, *KRT8*, *GATA2*, and *GATA3*, that were consistently highly expressed in TE and trophoblasts from day 6 through day 10 using our data (Fig 2F and 2H, and S1E Fig). Collectively, our single-cell RNA-seq data provided comprehensive transcriptomic profiling for trophoblast in in vitro cultured human conceptuses from blastocyst through early postimplantation stages.

### Weighted gene co-expression network analysis suggests genetic program dynamics for peri-implantation trophoblasts development

**Fig 3:**
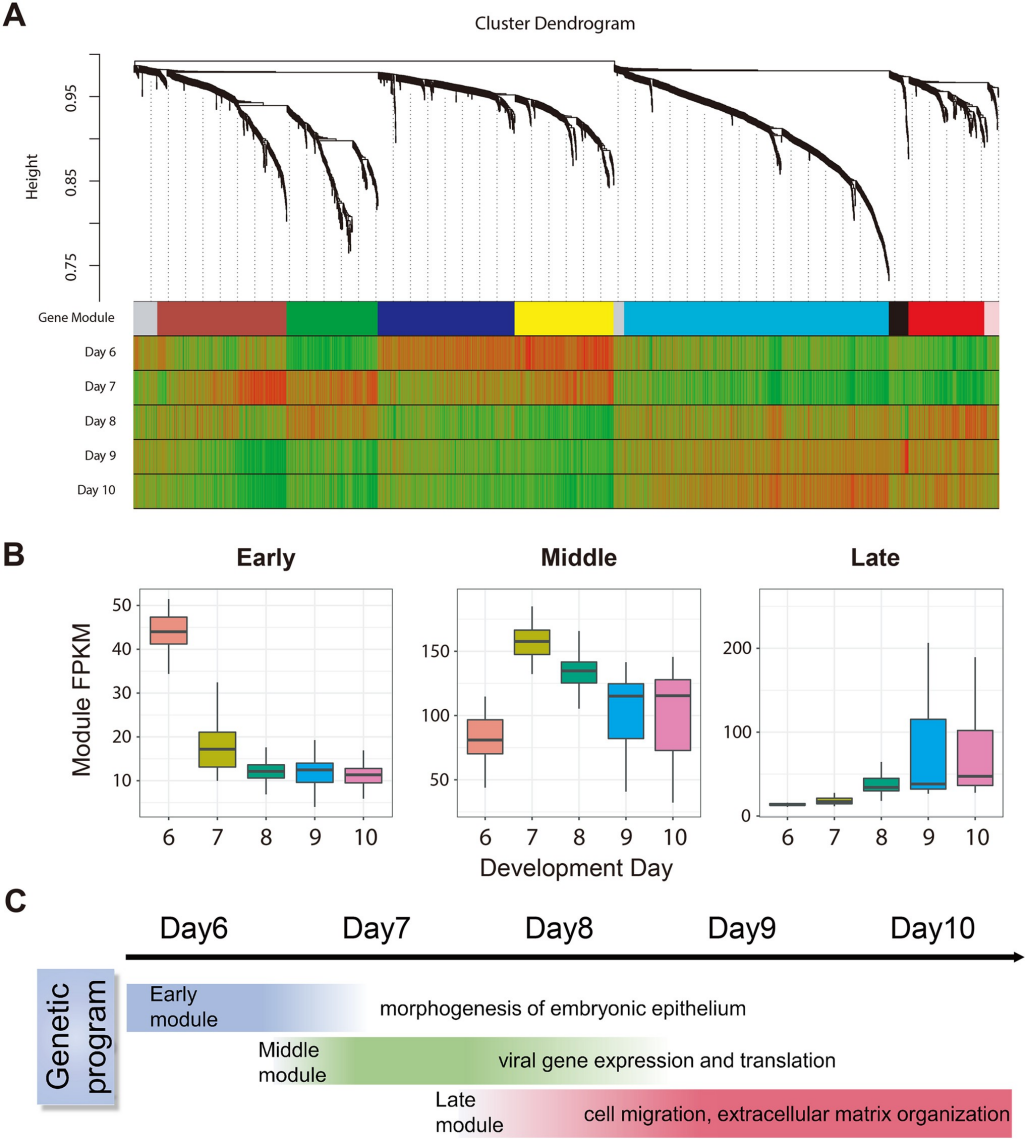
WGCNA suggested genetic networks related to trophoblast development. (A) Dendrogram showing the gene co-expression network constructed using WGCNA. The color bar labeled as “Gene Module” beneath the dendrogram represents the module assignment of each gene. The remaining color bars represent the correlation of genes with each developmental stage. Red means a gene is positively correlated with a developmental stage and therefore trend to be up-regulated at this stage; green means a gene is negatively correlated with a developmental stage and therefore trend to be down-regulated at this stage. Eight modules were classified into 3 genetic networks that activated at different developmental stages. (B) Box plots showing the distribution of network expression (mean FPKM of all genes within a given network) for different developmental stages. (C) Schematic drawing of the sequential transcriptome switches of representative GO pathways within each network. FPKM, fragments per kilobase of exon per million reads mapped; GO, gene ontology; WGCNA, weighted gene co-expression network analysis.

PCA showed that trophoblast cells were grouped by their development day (Fig 2D). To systematically investigate the genetic program dynamics, we performed weighted gene co-expression network analysis (WGCNA) on 2,464 genes that were variably expressed in trophoblast cells between different developmental stages. WGCNA identified 8 gene modules, each of which contains a set of genes that tend to be co-expressed at a certain development stage (Fig 3A). By relating module expression to development day, we found these 8 modules collectively represent 3 genetic networks that were specifically up-regulated at day 6, day 7 through 8 and day 8 through 10 (Fig 3B). These networks could represent core genetic programs that operate in the early (pre-implantation), middle (during implantation), or late (postimplantation) stages of trophoblasts development. We then performed gene ontology (GO) analysis of genes in each network to investigate their biological function. The early-stage network was enrichment in functional terms related to epithelium-like trophoblast development, including morphogenesis of embryonic epithelium, epithelial cell development, and epithelial tube formation. Human endogenous retrovirus (HERV)-associated genes are highly expressed in placenta and have an important function in ST formation [15]. The middle-stage network is significantly associated with RNA catabolic process, viral gene expression, and viral transcription, indicating *HERV*-associated genes are dynamically regulated at day 7 through 8. Genes in the late-stage network were specifically up-regulated in day 8 through 10. GO terms such as cell migration, extracellular matrix organization, and response to hypoxia were enriched in these genes, suggesting the activation of trophoblast invasion [16] (Fig 3C, S2 Fig, and S4 Table).

To further identify the genes that might play a critical regulatory role in these 3 genetic networks, we identified 240 hub genes based on the WGCNA measure of intramodular gene connectivity (kME; S5 Table). Hub genes are genes that centrally located within a gene module (kME > 0.8, *P* < 0.05) and have a co-expression relationship with many other genes and therefore could have critical regulatory functions. Intriguingly, the hub genes of 3 stages were enriched in GO terms similar to that of all WGCNA genes in corresponding stages, such as epithelial cell maturation for the early stage, viral transcription for the middle stage, and extracellular matrix organization for the late stage (S2 Fig, S4 and S5 Tables). We further found that many hub genes are related to critical placental function. For example, *EMP2* was a hub gene in the early-stage network. *Emp2*-deficiency in mice causes aberrant placental angiogenesis [17]. *ESRRG* was identified as a hub for the late-stage network, and abnormal reduction of *ESRRG* expression in human placenta is associated with intrauterine growth restriction and preeclampsia [18]. These results suggested WGCNA hub genes can be potential key regulatory genes for early placental development.

### Single-cell RNA sequencing revealed the timing of trophoblast differentiation

**Fig 4:**
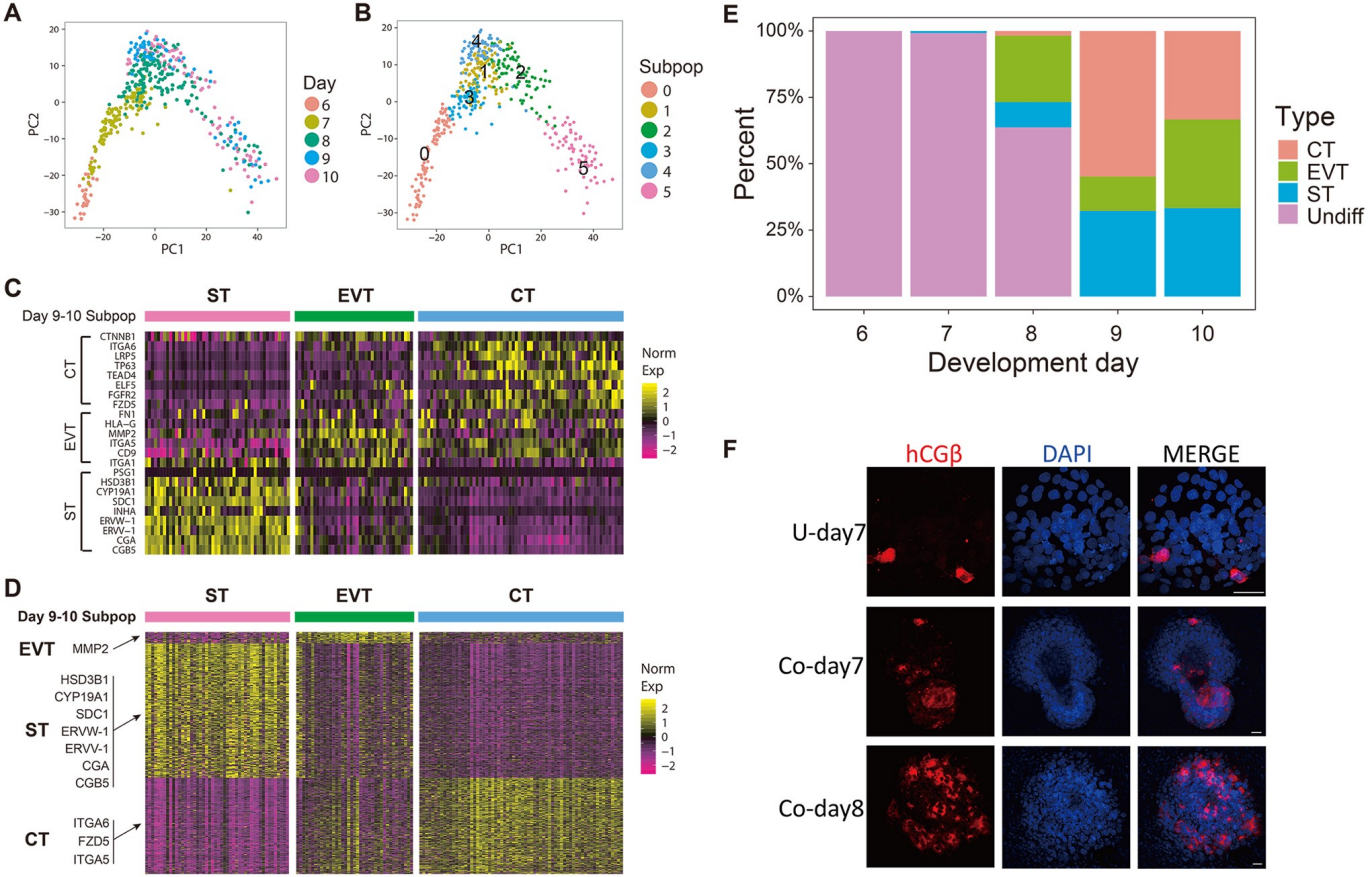
Single-cell RNA-seq revealed the timing of trophoblast differentiation. (A–B) Unbiased clustering based on SNN graph classified trophoblasts into 6 subpopulations. Cells on day 9 and day 10 were classified into 3 subpopulations. (C) Heat map showing the expression of previously defined ST, EVT, or CT marker genes within the 3 subpopulations identified from day 9 and day 10 cells. (D) Heat map showing the expression of genes that were specifically expressed in within the 3 subpopulations identified from day 9 and day 10 cells. We therefore annotated these 3 subpopulations as CT, EVT, and ST, respectively. (E) Stacked bar plot showing the percentage of the CT, EVT, and ST at different development days. The ST first appears at day 7 in cocultured conceptuses, whereas the EVT first appears on day 8. (F) Immunostaining of ST marker gene hCGβ in conceptuses at different stages (scale bars = 100 μm). CT, cytotrophoblast; EVT, extravillous trophoblast; RNA-seq, RNA sequencing; SNN, shared nearest neighbor; ST, syncytiotrophoblast.

Trophoblast sublineages such as EVT, CT, and ST are derived from multipotent trophoblasts. But when multipotent trophoblasts first differentiate into trophoblast sublineages is unclear [1]. To study this, we performed unbiased clustering using a shared nearest neighbor (SNN) graph-based clustering algorithm on 476 trophoblast cells and classified them into 6 subpopulations (Fig 4A and 4B, S3A and S3B Fig). Clusters 2, 4, and 5 together contain all cells from day 9, day 10, and a few cells from earlier days. By examining the expression of previously defined sublineages marker genes, we found that EVT markers (*ITGA5*, *HLA-G*, *FN1*, *MMP2*, *CD9*, and *ITGA1*) or ST markers (*CGB5*, *PSG1*, *HSD3B1*, *CYP19A1*, *SDC1*, *INHA*, *ERVW-1*, *ERVV-1*, and *CGA*) highly expressed in Cluster 2 or 5, whereas Cluster 4 co-express CT markers (*ITGA6*, *TP63*, *CTNNB1*, *LRP5*, *TEAD4*, *ELF5*, *FGFR2*, and *FZD5*) and EVT marker genes similar to Cluster 1 [19–22] (Fig 4C). We then identified genes that were specifically expressed in Clusters 2, 4, and 5. We found that many ST marker genes, such as *HSD3B1*, *CYP19A1*, *SDC1*, *ERVW*-1 (*Syncytin*-1), *ERVV*-1, *CGA*, and *CGB*, were specifically highly expressed in Cluster 5. CT markers, such as *ITGA6* and *FZD5*, were specifically expressed in Cluster 4. A few EVT marker genes such as *MMP2* were specifically highly expressed in Cluster 2 (Fig 4D). In addition, we found that genes specifically up-regulated in Cluster 5, Cluster 4, and Cluster 2 are significantly enriched in ST, CT, and EVT marker genes identified using bulk RNA-seq data of primary cells (Hypergenomic test, *p* = 6.644473 × 10^−7^ for EVT, *p* < 2.225074 × 10^−308^ for ST, 1.776357 × 10^−15^ for CT). Taken together, these results suggest that Cluster 5, Cluster 4, and Cluster 2 represent ST, CT, and EVT, respectively. The rest of the clusters consist of trophoblasts from day 6 through day 8 and express CT markers. These results indicated that these clusters represent multipotent trophoblasts that have not committed to differentiation.

We then tried to determine when EVT and ST were established in conceptuses. We found that ST first appears in cocultured conceptuses at day 7, even though at a very low percentage (1 out of 60 cells). ST cells then become more abundant at day 8 (16 out of 168 cells; Fig 4E, S3 Table). Immunostaining showed that hCGβ positive cells can be detected as early as day 7 and become more abundant at day 8 (Fig 4F). These results suggested that ST cells first occur after day 7 and become more abundant after day 8. Similarly, we found EVT cells were absent in all the day 7 conceptuses but appear at day 8, indicating EVT were generated after day 7 (Fig 4E, S3C Fig). Taken together, our results revealed the time course when ST and EVT appear in peri-implantation conceptuses in vitro.

### Single-cell bifurcation analysis using the variance of gene expression identifies TBX3 as a novel required regulator for trophoblast differentiation

The ST is an important trophoblast sublineage that forms the primary barrier between maternal and fetal circulation and synthesizes hormones vital for pregnancy. The ST is derived from multipotent trophoblasts within TE and CT. Previous studies using mature placentas and cell lines have demonstrated that many regulatory factors and pathways have been reported to be linked with the human ST formation [23–26]. However, these results must be interpreted carefully, because these in vitro differentiation models may not perfectly recapuliate the mechanism for trophoblast differentiation at peri-implantation stage.

Our data provided a unique opportunity to study how trophoblast differentiation is regulated in vivo, especially during early placenta development. It is generally accepted that the mechanism underlying a cell-fate decision event can be summarized as a hierarchical model: a small number of “upstream” regulators, such as transcriptional factors, were activated in a subset of cells before cell-fate decision. These upstream regulators then activate a larger number of cell-type specific genes, which initiate the fate transition and confer cell-type specific function. The identification of the upstream regulator can be a critical step for understanding the molecular mechanism controlling the trophoblast differentiation. However, the lack of a computational method that can systematically identify upstream regulators underlying a cell-fate decision event poses a major challenge for our analysis.

To identify the upstream regulatory genes that drive the cell-fate transition from multipotent trophoblast to ST, we designed single-cell bifurcation analysis using variance of gene expression (SCBAV), a method derived from single-cell clustering using bifurcation analysis (SCUBA) [27]. Our method is a computational strategy that can systemically screen for upstream regulators using time-serial single-cell RNA sequencing (scRNA-seq) data (S4A Fig). Briefly, SCBAV first reconstructs a cell development trajectory from scRNA-seq data. A cell-type transition will be represented as a bifurcation event in the trajectory. SCBAV then screens the upstream regulator underlying a bifurcation event according to gene expression level and variation. Transcription factors that are highly variable before bifurcation and significantly differentially expressed in 2 lineages after bifurcation are likely to be the upstream regulators. By applying SCBAV on our trophoblast cells data set, we identified a bifurcation event that happens after day 8 (S4B and S4C Fig). CT and EVT markers were highly expressed in one lineage after bifurcation, whereas ST markers were greatly up-regulated in the other lineage (S4D–S4I Fig). This bifurcation, therefore, captured the cell-fate segregation of ST from CT and EVT. SCBAV found 26 putative upstream regulators driving this bifurcation. Among them, TBX3 is the only transcription factor and was ranked as the most top by both of 2 upstream regulator screening criteria (S4J–S4L Fig). Consistent with those results, immunostaining shows that TBX3 was highly expressed in day 8 and day 10 conceptuses (S5A Fig). In addition, ST cells showed higher TBX3 expression level compared with EPI, PE, and non-ST trophoblast cells (S5B–S5D Fig) [28]. Taken together, these results suggested TBX3 could be important in controlling trophoblast differentiation into ST.

### TBX3 is required for development of ST

**Fig 5:**
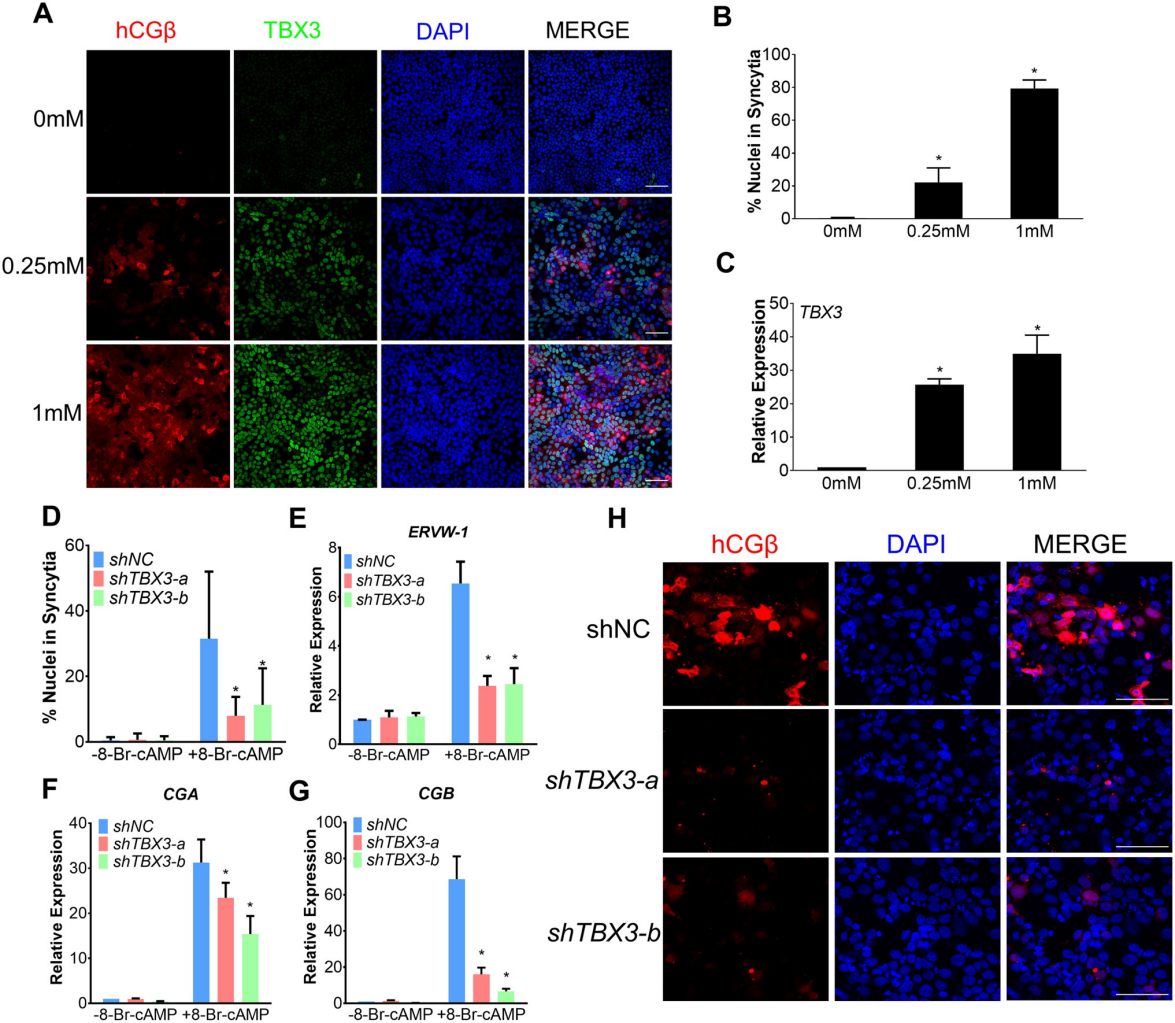
TBX3 regulated trophoblast cell differentiation into ST. (A) Immunofluorescence images of hCGβ (red) and TBX3 (green) showing ST cells formation under 0 mM, 0.25 mM, and 1 mM 8-Br-cAMP for 48 hours. (B) Graphical depiction of the percentage of nuclei associated with ST cells following exposure to different concentrations of 8-Br-cAMP for 48 hours. (C) qRT-PCR for *TBX3* expression following exposure to different concentrations of 8-Br-cAMP for 48 hours. (D) The percentage of nuclei associated with fused cells. (E–G) qRT-PCR for *ERVW*-1 (E), *CGA* (F), and *CGB* (G) expression in JEG-3 cells expressing *shNC*, *shTBX3-a*, or *shTBX3-b* before and after 0.25 mM 8-Br-cAMP treatment for 48 hours. (H) Representative images of hCGβ expression in JEG-3 cells expressing *shNC*, *shTBX3-a*, or *shTBX3-b* cultured under 0.25 mM 8-Br-cAMP for 48 hours. Underlying data for all panels included in this figure can be found in S1 Data. *p < 0.05, *n* ≥ 3, mean ± SD. (scale bars = 100 μm.) cAMP, cyclic adenosine monophosphate; qRT-PCR, quantitative real-time PCR; ST, syncytiotrophoblast; TBX3, T-box transcription factor 3.

To validate the function of TBX3 in trophoblast differentiation, we used a well-established in vitro trophoblast differentiation system, which modeled CT-to-ST differentiation by treating trophoblast cell line JEG-3 with cyclic adenosine monophosphate (cAMP) analog 8-Br-cAMP. The generation of ST can be characterized by cell fusion and ST marker genes expression [25, 29]. In control JEG-3 cells that were not treated by 8-Br-cAMP, the cell fusion ratio was less than 1%, and ST marker hCGβ expression was almost undetectable, indicating ST generation before treatment is minimal (Fig 5A and 5B, 0mM group). After 48 hours treatment with 8-Br-cAMP, JEG-3 cells exhibited greatly enhanced cell fusion and hCGβ compared with the control group, and the fusion ratio is correlated with 8-Br-cAMP concentration (Fig 5A and 5B). Notably, TBX3 was not expressed in the control group but was significantly up-regulated by 8-Br-cAMP treatment, and the up-regulation fold is also correlated with 8-Br-cAMP concentration (Fig 5A and 5C).

We then knocked down TBX3 in JEG-3 cells using lentiviral vectors expressing TBX3 short hairpin RNA (shRNA) and assessed its influence on ST generation. Control shRNA (*shNC*) targeting no known mammalian RNA and shRNA targeting TBX3 (*shTBX3*-a and *shTBX3*-b) had no discernible influence on proliferation of JEG-3 cells. However, *shTBX3*-a and *shTBX3*-b interference resulted in 96% ± 1% and 97% ± 1% suppression of *TBX3* mRNA levels after 8-Br-cAMP treatment, respectively, and also repress TBX3 protein levels (S6A and S6B Fig). TBX3 knockdown also significantly reduced cell fusion (75% ± 7% and 64% ± 14% reduction in *shTBX3*-a and *shTBX3*-b cells, respectively; Fig 5D) and down-regulated ST markers transcription, including human chorionic gonadotropin subunits (*CGA* and *CGB*), *Syncytin* (*ERVW*-1), and other HERV-derived genes (*ERVV*-1 and *ERVV*-2; Fig 5E–5G, S6C Fig). Immunostaining showed that the hCGβ protein level was decreased by TBX3 knockdown (Fig 5H). Taken together, these results demonstrated that TBX3 is required for ST formation.

### Coculturing with EM cells influences genes related to trophoblast development

PCA showed that u-day 7 trophoblasts do not overlap with co-day 7 trophoblasts (S7A Fig). Consistently, pseudotime analysis showed that developmental stages of u-day 7 and co-day 7 trophoblasts are significantly different (S7B Fig). These results indicate that EM cells have a profound impact on gene expression in trophoblasts. To systematically dissect the influence of coculturing, we identified the differentially expressed genes (DEGs) between co-day 7 and u-day 7 trophoblasts. We found 241 genes were significantly up-regulated in co-day 7 cells, and 140 genes were significantly down-regulated in co-day 7 cells (Wilcox test, adjusted *p* < 0.05; S6 Table). GO enrichment analysis showed that genes up-regulated in co-day 7 cells are associated to terms such as mRNA processing and translation, whereas down-regulated genes are associated to terms including skeletal muscle tissue regeneration and epithelial cell maturation (S6 Table). By examining the DEGs, we found *EIF5A*, a gene involved in trophoblast proliferation, migration, and invasion; *WEE1*, a gene regulating mitosis associated with cell cycle progression in trophoblast cells; and *CCR7*, a chemokine gene associated with trophoblast differentiation, were significantly up-regulated by coculturing in day 7 trophoblasts [30–32]. On the other side, *MDM2*, a gene associated with preeclampsia susceptibility, was significantly down-regulated by coculturing [33]. These results suggest that coculturing with EM cells can influence the expression of genes related to trophoblast development.

### Transcriptomic analysis reveals expression pattern of polar-TE marker genes in peri-implantation trophoblast subpopulations

**Fig 6:**
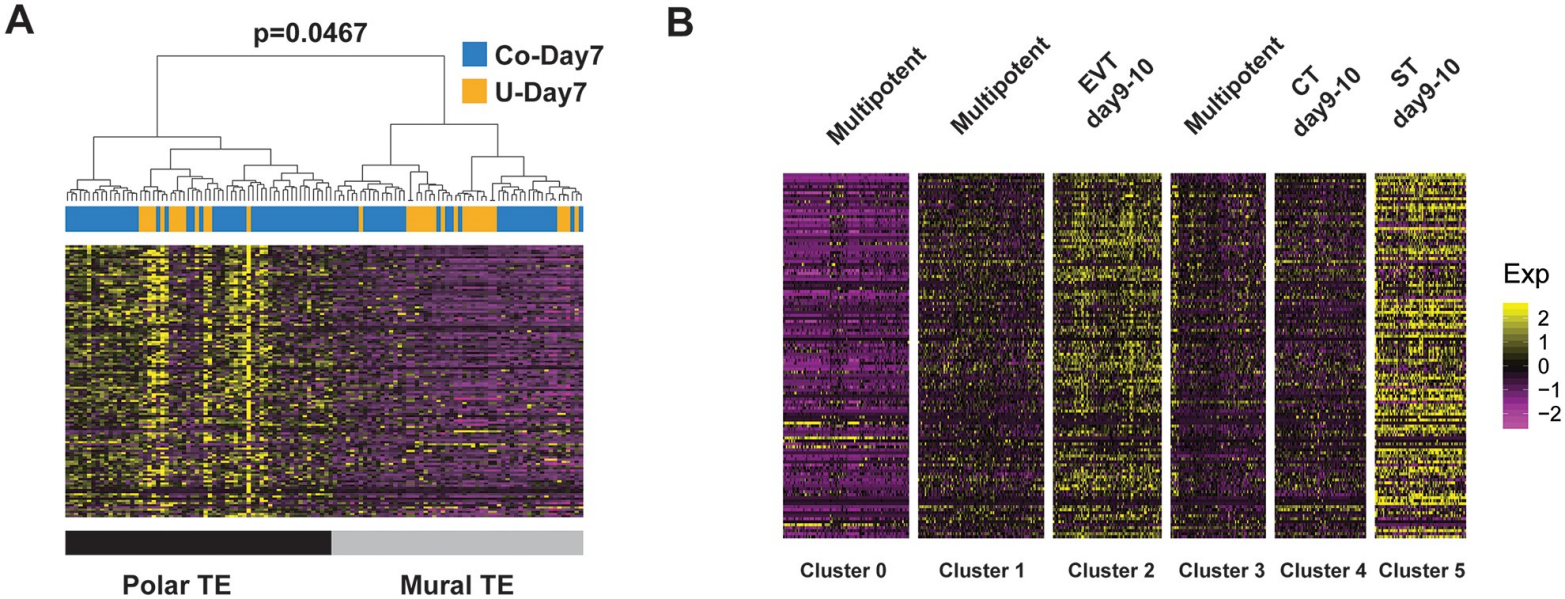
Transcriptomic analysis of polar-TE marker genes. (A) Hierarchical clustering using previously identified polar-TE markers classified day 7 trophoblasts into 2 clusters with high or low polar-TE markers expression, respectively. (B) Heat map showing the expression of previously identified polar-TE markers at 6 trophoblast clusters identified before. CT, cytotrophoblast; EVT, extravillous trophoblast; ST, syncytiotrophoblast; TE, trophectoderm.

Polar and mural TE cells are TE subpopulations that emerge at the blastocyst stage [14, 34]. Previous studies showed that most of the human blastocysts attach to endometrium at the polar side, and polar TEs first proliferate and invade into endometrium [1, 2]. These published studies together implicated the polar TE could have an important function for development, especially implantation. But the role of polar TE during postimplantation development is still elusive. We therefore sought to study this question using our scRNA-seq data. Hierarchical clustering using 129 previously identified polar-TE markers [14] robustly separate day 7 trophoblast cells into 2 subpopulations, which is consistent with the existence of polar and mural TE at day 7 (Fig 6A). We then sought to investigate the relationship between polar and mural TE and the 6 subpopulations we identified above. We found that polar-TE markers were lowly expressed in all day 6 cells and day 7 non-cocultured cells and moderately expressed in CT cells at different developmental days. Interestingly, polar-TE markers were significantly up-regulated in differentiated trophoblasts, including EVT and ST (Fig 6B, S8 Fig). These results showed that most of the polar-TE markers were up-regulated in differentiated trophoblast cells.

## Discussion

**Fig 7:**
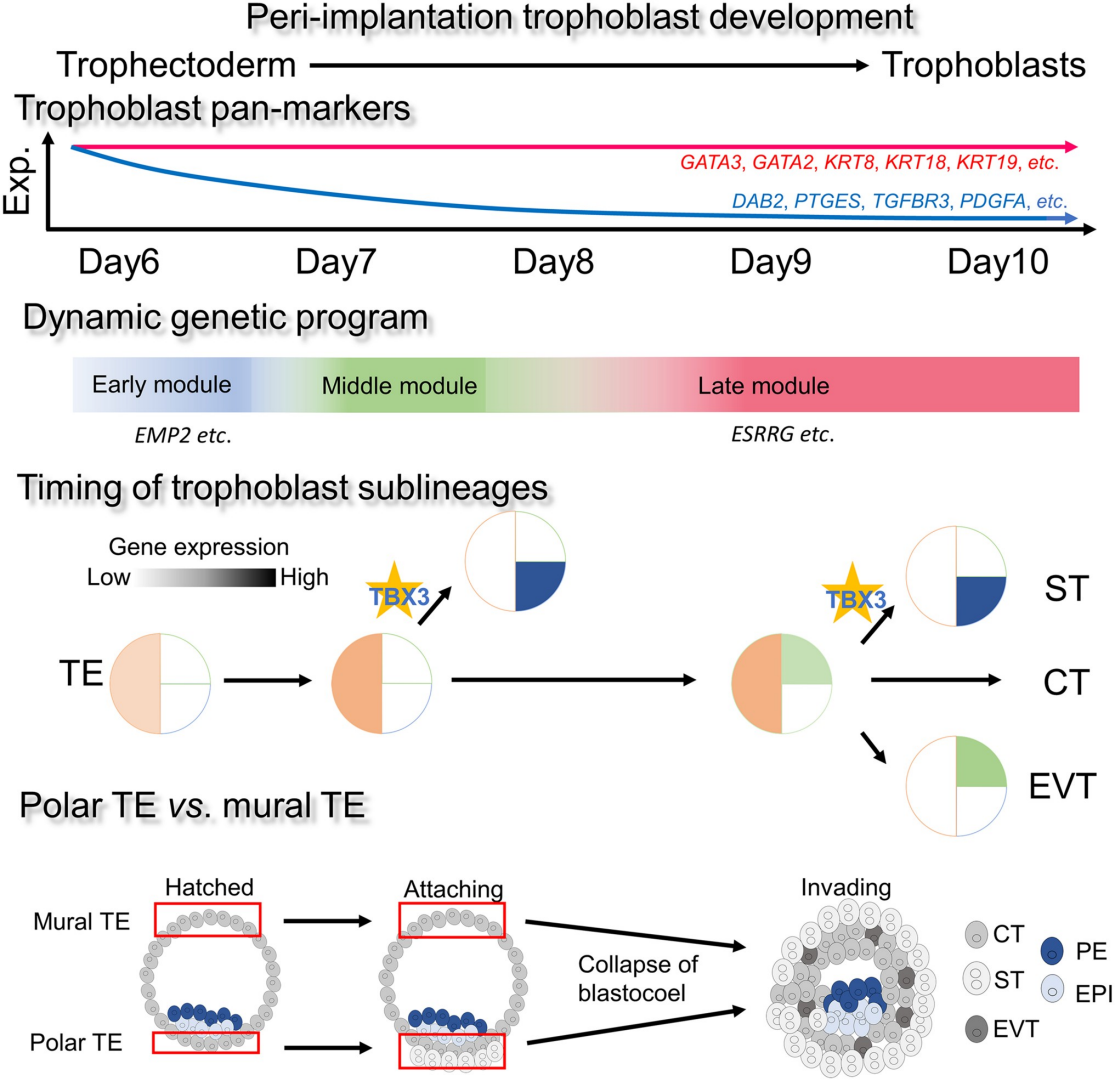
Schematic diagram of trophoblast development during peri-implantation. Our single-cell profiling of peri-implantation trophoblasts revealed the differential expression of trophoblast pan-markers and the dynamic genetic program of trophoblast development. ST cells were abundant at day 8, and TBX3 was critical for ST formation using SCBAV as well as in vitro verification. Transcriptome analysis showed that most of polar-TE markers were up-regulated in differentiated trophoblast cells. CT, cytotrophoblast; EPI, epiblast; EVT, extravillous trophoblast; PE, primitive endoderm; SCBAV, single-cell bifurcation analysis using variance of gene expression; ST, syncytiotrophoblast; TBX3, T-box transcription factor 3; TE, trophectoderm.

Trophoblasts undergo magnificent morphological movement and cellular changes after implantation. In this study, by profiling over 500 single cells in 19 conceptuses generated using a coculture system, we reconstruct the transcriptome dynamics of trophoblasts through blastocyst to early postimplantation stages (Fig 7). Our study complements previous studies that use scRNA-seq to profile trophoblasts and other cell types within mature placenta [4–7]. However, it should be noted that although the in vitro human conceptus coculture model can recapitulate the events of cellular differentiation of TE lineage, it cannot fully represent the postimplantation development of conceptuses because of the limited development of EPI compartment [10–13].

Nonhuman mammals have been continuously used as models to study pregastrulation development. Although similar sets of transcription factors, including POU class 5 homeobox 1 (POU5F1), SRY-box transcription factor 2 (SOX2), and Nanog homeobox (NONOG), drive inner cell mass (ICM) lineage formation in most mammalian species, the expression pattern and function of transcription factors related to peri-implantation trophoblast development can be quite different among different species (Fig 2F) [11, 12, 14, 35–37]. For example, caudal type homeobox 2 (Cdx2) is one of the main transcription factors that controls self-renewal, and differentiation begins to express at early-stage blastocysts in murine. But it is not expressed in primate embryos until peri-implantation stages [11, 12, 35, 38]. These results suggest TE development is divergent between species. In addition, although murine blastocysts invade into the endometrium at the mural TE, the primate blastocysts usually attach to the endometrium at the side of the polar TE [34, 39]. Previous studies have identified genes that are highly expressed in polar TE compared with mural TE in human blastocysts[14, 40]. In our study, we found that most of these 129 previous identified polar-TE markers are up-regulated in differentiated trophoblasts (EVT and ST; Fig 6B, S8 Fig), compared with multipotent trophoblasts. Our results therefore highlight the difference between species, in terms of TE polarity.

Our results suggest TBX3 has a critical role in regulating the ST formation. TBX3 haploinsufficiency in human causes ulnar-mammary syndrome (UMS), a genetic disorder characterized by abnormal forelimb and apocrine gland development [41]. Although *TBX3* is abundant in human placenta [42], its role in the placenta has not been studied. Our results demonstrated that TBX3 is required for both hCGβ and syncytium formation, indicating TBX3 is an upstream regulator for ST formation.

HERV-derived genes are up-regulated in ST, and their expression is regulated by DNA methylation [43]. Using the in vitro trophoblast differentiation system, we found that *DNMT1* and *DNMT3B* are significantly down-regulated after 8-Br-cAMP treatment, whereas *TET2* and *TET3* are significantly up-regulated after 8-Br-cAMP treatment (S6C Fig). These results are consistent with our scRNA-seq data and indicate that *DNMT1*, *DNMT3B*, *TET2*, and *TET3* might be involved in the regulation of DNA methylation on HERVs during trophoblast differentiation. However, TBX3 knockdown down-regulated *DNMT1* but did not significantly influence the expression level of *DNMT3B*, *TET2*, or *TET3* (S6C Fig). These results indicate that TBX3 can regulate HERV expression but not through altering the transcription level of *DNMT*s and *TET*s. Further studies should be done to elucidate how TBX3 regulates HERV expression in trophoblasts.

Our scRNA-seq data revealed that *DPPA3* (also known as *STELLA* and *PGC7*) expression is significantly down-regulated in ST compared with CT and EVT (S9 Fig). Consistently, in the trophoblast in vitro differentiation system, *DPPA3* is significantly down-regulated by 8-Br-cAMP treatment, whereas TBX3 knockdown does not significantly alter *DPPA3* expression (S6C Fig). Because Dppa3 is a factor that safeguards DNA methylation at ERVs in mouse embryos and involves pluripotency circuit in mouse embryonic stem cell (mESC) [44], these results suggest DPPA3 in the human could maintain differentiation potential of trophoblast progenitors and repress trophoblast differentiation and deactivating HERVs and HERV-derived genes.

In conclusion, our study established single-cell transcriptomic profiles of peri-implantation trophoblast cells. Moreover, we have found a new role of TBX3 as a “required upstream” regulator of the trophoblast differentiation in the human. Our study offers unique resources for understanding the early placenta development and pathogenesis associated with early trophoblast defects.

## Materials and methods

### Ethics statement

All the embryos and endometrium tissue donors signed informed consent, as detailed in the formal informed consent forms in Chinese and informal English translation in S1 Text. All the procedures were approved by the Institutional Review Board (IRB) of Tongji Hospital in Tongji University (KYSB-2017-072).

### Human conceptus culture and isolation

The embryos were voluntarily donated by the patients at the Center for Clinical Reproductive Medicine at Tongji Hospital of Tongji University with informed consent and institutional approval. Human peri-implantation conceptuses were cultured according to protocols [11, 12] with the addition of endometrium cells. When blastocysts hatch out from zona pellucida at day 6.5, we transferred blastocysts to dishes plated with human EM cells. Non-coculture day 7 conceptuses were used as a control group to coculture day 7. Single cells of conceptuses randomly collected from all stages were acquired according to a previous procedure by Xue and colleagues [45]. For attached conceptuses from day 7 to day 10, they underwent short incubation in trypsin to detach from dishes and were manually transferred to a new dish containing trypsin for further dissociation for 3 to 5 minutes with repeated aspiration using a mouth-operated, drawn capillary pipette. A single cell was then manually picked using the capillary pipette into a 0.2-ml PCR tube containing lysis buffer.

### Single-cell RNA-seq library preparation

Single-cell RNA-seq was performed using SMART-seq2 protocol with minor modification [46, 47]. Briefly, a single cell was first lysed in 0.5 μL lysis buffer, followed by reverse transcription using Superscript III. After purification using Ampure XP Beads, amplified cDNA product was diluted to 0.1 ng/μL. A total of 0.1 ng amplified cDNA was used for library construction. Libraries were pooled and sequenced on Illumina Hiseq X10 in paired-end, 150 bp mode.

### Single-cell RNA-seq data processing

Single-cell RNA-seq data were first trimmed with TrimGalore! using following parameters “-q 20—phred33—gzip—length 30—paired” to remove adaptor sequences and low-quality bases. The trimmed data were then aligned to human reference genome hg38 using STAR version 2.6.0 in the pair-end mode with default parameters. The number of reads mapped to each gene was counted using featureCounts version 1.6.2 and Gencode hg38 gene annotation. Customized R scripts and published R packages, including Seurat, were used in subsequent analysis.

### Identification of TE-, EPI-, and PE-lineage cells and identification of peri-implantation trophoblast markers

To our experience, the majority of cells in peri-implantation conceptuses are of trophoblast lineage. Therefore, random picking ensures us to unbiasedly sample trophoblast population with a few cells of EPI or PE lineage. Conceptus cells were assigned into 3 lineages, namely, TE, EPI, and PE, based on their expression of 300 previous identified lineage marker genes. Specifically, the read-count matrix was first normalized and quality-controlled using Seurat. The lineage identity for each cell was then determined using a previous strategy reported before by Butler and colleagues [48]. Briefly, for each cell, a “TE score,” an “EPI score,” and a “PE score” were computed using AddModuleScore function implemented in Seurat package, based on its expression of previously identified markers for each lineage, respectively. The cell lineage was then defined as the lineage that had the highest score. To identify maintained trophoblast markers, we started with previously identified TE marker genes for pre-implantation conceptuses and excluded genes that were lowly expressed in trophoblasts at any stage between day 6 to day 10 (mean FPKM < 10 overall trophoblasts on each day).

### WGCNA

WGCNA was performed on normalized gene expression data measured in read count per million (RPM) metric, using 2,464 highly variably expressed genes determined by FindVariableGenes function in Seurat. The WGCNA was then performed following the previously published study by Xue and colleagues [45]. Briefly, the topological overlap matrix (TOM) was constructed with softPower and was set to 8. The hub genes for each module were identified as module eigengene based connectivity kME > 0.8 and *P* < 0.05. The GO enrichment analysis was performed using GO Consortium website (http://geneontology.org) and R package clusterProfiler.

### Single-cell pseudotime analysis

Single-cell pseudotime analysis was performed using principal curve method, as described before by Guo and colleagues [49]. Briefly, highly variable genes were identified, and PCA was performed using these highly variable genes with Seurat packages. The first 2 principal components were used to fit principle curve, using R package “pcurve.” The inferred principle curve can represent the single-cell developmental trajectory, and by computing the projection of each cell to the principle carve, the pseudotime of each cell can be determined.

### SCBAV

SCBAV consists of 2 steps. It first constructs a cell lineage trajectory using scRNA-seq data and identifies when lineage segregation happens. It then identifies the upstream regulator that could cause the lineage segregation based on certain criteria. Briefly, SCBAV represents all cells from different time points on the same reduced dimension using PCA. The first *N* principal components are used, such that *N* is the smallest number that can capture at least 40% of the total variation within the data. Next, within this reduced dimension, SCBAV identifies cell subpopulations within cells from each time point, using Gap Statistics and K-means clustering. The Gap Statistics is used to determine the number of subpopulations within cells from each time point, and K-means clustering is used to classify cells into clusters based on the cluster number determined by Gap Statistics. SCBAV then constructs cell lineage trajectory by iteratively connecting the most similar clusters between 2 time points. Specifically, for a Cluster A in time point t, SCBAV connects it to the cluster in t+1, whose centroid as the highest correlation with Cluster A’s centroid among all clusters in t+1. SCBAV keeps doing this until all clusters from all time points were connected to one lineage trajectory.

A cell-fate segregation event will be represented as a bifurcation on the cell-fate trajectory. Suppose there is a bifurcation event in the lineage trajectory right after time point t. Bifurcation event represents a cellular differentiation event. SCBAV then tries to identify the upstream regulator that drives the bifurcation. According to the hierarchical model for cell-fate transition regulation mentioned above, an upstream regulator that promotes a cell lineage bifurcation must meet 2 criteria: (1) it is consistently differentially expressed between 2 lineages after bifurcation point; (2) it is highly variably expressed in the cell population right before bifurcation.

### Cell culture and shRNA constructs

Endometrium tissue was obtained from patients at Tongji Hospital of Tongji University. The endometrium tissue donor with no genetic disorder or diseases signed informed consents. Primary endometrium was dissected using scalpels and then enzymatically digested in 1% collagenase I and IV (Gibco, Grand Island, NY, 17100–017 and 17014–019) with repeated pipetted up and down for 30 minutes at 37 °C. After filtration through 40 μm strainers and centrifugation at 300*g* for 3 minutes, the supernatant was removed, and the pellet at the bottom was washed with PBS twice. Then, the pellet was resuspended and prepared for use.

JEG-3 trophoblast cells were purchased from Cell Bank of Chinese Academy of Sciences and were cultured in MEM medium (Gibco, Grand Island, NY, 10370021) with the addition of 0.11 g/L Sodium Pyruvate (Gibco, Grand Island, NY, 11360070) and 10% FBS (Gibco, Grand Island, NY, 10099141). To stimulate JEG-3 trophoblast differentiation, cells were treated with 8-Br-cAMP (Sigma-Aldrich, St. Louis, MO, B7880).

Human *TBX3* shRNA and control shRNA constructs in pLenR-GPH vectors were purchased from Shanghai Taitool Bioscience Co., Ltd. (China). The target sites for the shRNAs are *TBX3*-a, GCGAATGTTTCCTCCATTTAA; *TBX3*-b, GCAGTCCATGAGGGTGTTTGA; Control, TTCTCCGAACGTGTCACGT. Lentiviruses were also generated in Shanghai Taitool Bioscience Co., Ltd. (China). For *TBX3* knockdown experiment, lentivirus transduction at a multiplicity of infection (MOI) of 10 was accomplished according to previous studies [50, 51]. Transfected Cells were treated with the addition of 2 μg/mL puromycin (Gibco, Grand Island, NY, A1113803) for 2 to 3 passages, and cells stably expressing GFP were used for subsequent analyses.

### Immunofluorescence staining

Conceptuses and cells growing on coverslips were fixed in 4% paraformaldehyde and were permeabilized in 0.2% triton X-100 for 10 minutes. Subsequently, conceptuses and cells were blocked at room temperature in 5% BSA in PBS for 1 hour and incubated with primary antibodies (hCGβ, Abcam, United Kingdom, ab9582; TBX3, Abcam, United Kingdom, ab99302; HLA-G, Abcam, United Kingdom, ab52454; OCT4, Santa Cruz Biotechnology, Santa Cruz, CA, sc-5279) in blocking solution overnight at 4 °C. The conceptuses and cells were then washed twice in blocking solution and incubated with species-appropriate fluorescent-conjugated secondary antibodies at room temperature for 1 hour before final washes in blocking solution. Coverslips with conceptuses or cells were then moved to drops of Vectashield mounting media with DAPI (Vector Lab, United Kingdom, H-1200) on slides for 10 minutes of incubation before imaging. For non-cocultured conceptuses, all immunostaining operations were under the stereoscopic microscope. Cell fusion index was analyzed according to previous reports [25, 29].

### qRT-PCR

Total RNA was extracted from cultured cells using Takara MiniBest Universal RNA Extraction Kit (Takara, Japan, 9767). cDNA was synthesized through RevertAid First Strand cDNA Synthesis Kit (Thermo Scientific, Waltham, MA, K1622). qRT-PCR was carried out using TB Green Premix Ex Taq II (Takara, Japan, RR820A) and Roche Light Cycler 96 system (Roche, Switzerland). Relative levels of RNAs were calculated by the ddCt method with *GAPDH* as endogenous controls. The primers were shown in S7 Table.

## Supporting information

S1 Fig Scatter plot of TE-lineage markers expression identified previously and in our study. (A) PCA showing the unbiased clustering of day 6 through day 10 trophoblast cells, using all detected genes. (B) EPI-, PE- or TE-lineage cells were annotated based on Fig 2C. Cell identities were visualized on PCA related to Fig S1A. (C) Heat map showing the average expression of previously proposed markers among EPI, PE, and TE in 3 lineages. (D) Scatter plot showing the expression of previously identified TE-lineage markers. (E) Scatter plot showing the expression of highly expressed TE-lineage markers identified in this study. EPI, epiblast; PCA, principle component analysis; PE, primitive endoderm; TE, trophectoderm.
(TIF)

S2 Fig Bobble plot of the GO enrichment of genes in each network. GO, gene ontology.
(TIF)

S3 Fig Single-cell RNA-seq revealed the clusters of trophoblasts across all development days. (A) Stacked bar plot showing the parentage of cells of 6 subpopulations at different development days. (B) Heat map showing the expression of previously identified CT, EVT, and ST markers in 6 trophoblast subpopulations. (C) Immunostaining of HLA-G in day 7 and day 8 conceptuses. (Scale bars = 100 μm.) CT, cytotrophoblast; EVT, extravillous trophoblast; HLA-G, human leukocyte antigen-G; RNA-seq, RNA sequencing; ST, syncytiotrophoblast.
(TIF)

S4 Fig SCBAV identified TBX3 as a novel upstream regulator for trophoblast differentiation. (A) Graphical abstract of SCBAV. (B) Cell trajectory reconstructed by SCBAV. (C) The bifurcation within the SCBAV cell trajectory recapitulated the cell-fate divergence of ST from CT and EVT. (D–F) Expression of ST specific genes within 2 lineage branches. (G–I) Expression of CT specific genes within 2 lineage branches. (J–L) TBX3 is variably expressed before bifurcation point and significantly up-regulated in ST compared with EVT and CT after bifurcation. CT, ytotrophoblast; EVT, extravillous trophoblast; SCBAV, single-cell bifurcation analysis using variance of gene expression; ST, syncytiotrophoblast; TBX3, T-box transcription factor 3.
(TIF)

S5 Fig The expression of TBX3 in the conceptuses. (A) Immunostaining of hCGβ and TBX3 in day 8 and day 10 conceptuses. Scale bars = 100 μm. (B) Immunostaining of OCT4 and TBX3 in day 8 and day 10 conceptuses. Scale bars = 50 μm. (C–D) Violin plot showing the expression of TBX3 in 3 conceptus lineages (C) and in different TE subtypes (D). OCT4, alias of POU class 5 homeobox 1 (POU5F1); TBX3, T-box transcription factor 3; TE, trophectoderm.
(TIF)

S6 Fig TBX3-regulated trophoblast cell differentiation. (A) and (C) qPCR for *TBX3*, *ERVV*-1, *ERVV*-2, *DNMT1*, *DNMT3a*, *DNMT3B*, *TET1*, *TET2*, *TET3*, and *DPPA3* expression in JEG-3 cells expressing shNC, *shTBX3*-a, or *shTBX3*-b before or after 0.25 mM 8-Br-cAMP treatment for 48 h. *p < 0.05, *n* ≥ 3, mean ± SD. (B) Representative images of TBX3 expression in JEG-3 cells expressing shNC, *shTBX3*-a, or *shTBX3*-b cultured under 0.25 mM 8-Br-cAMP for 48 h. Underlying data for all panels included in this figure can be found in S1 Data. (Scale bars = 100 μm.) cAMP, cyclic adenosine monophosphate; qPCR, quantitative PCR; shNC, short hairpin negative control RNA; TBX3, T-box transcription factor 3.
(TIF)

S7 Fig Coculturing induced transcriptomic changes related to trophoblast development. (A) PCA showing the 6 subpopulations within all trophoblasts. (B) Violin plot showing the pseudotime distribution of trophoblast population under different culture condition across different development days. The pseudotime of each cell is inferred using principal curve analysis. PCA, principle component analysis.
(TIF)

S8 Fig Box plot showing the distribution of average expression of polar-TE markers among 6 trophoblast subpopulations. The average expression of polar genes are significantly higher in Cluster 2 (EVT day 9–10, *p* < 2.2 × 10^−16^) and Cluster 5 (ST day 9–10, *p* = 1.64 × 10^−14^) compared with other clusters (CT and multipotent trophoblasts). CT, cytotrophoblast; EVT, extravillous trophoblast; ST, syncytiotrophoblast; TE, trophectoderm.
(TIF)

S9 Fig Genes expressed differentially in peri-implantation trophoblast lineages. (A–B). Scatter plot and violin plot showing the expression of upstream regulators, ST marker genes, DNA methyltransferases, and TET methylcytosine dioxygenases. ST, syncytiotrophoblast; TET, ten-eleven translocation.
(TIF)

S1 Table Summary of TE, EPI, and PE cells across development days. EPI, epiblast; PE, primitive endoderm; TE, trophectoderm.
(DOCX)

S2 Table Statistical analysis of *DAB2*, *PTGES*, *TGFBR3*, and *PDGFA* expression between day 6 and day 7. (DOCX)

S3 Table Summary of 6 trophoblast clusters across development days. (DOCX)

S4 Table Excel spreadsheet containing GO analysis of early, middle, and late module genes of WGCNA. GO, gene ontology; WGCNA, weighted gene co-expression network analysis.
(XLSX)

S5 Table Excel spreadsheet containing 240 hub genes and GO analysis of hub genes in the early, middle, and late module. GO, gene ontology.
(XLSX)

S6 Table Excel spreadsheet containing DEGs of co-day versus u-day trophoblast cells and GO analysis of differentially expressed genes. DEG, differentially expressed gene; GO, gene ontology.
(XLSX)

S7 Table Primers used for qRT-PCR. qRT-PCR, quantitative real-time PCR.
(DOCX)

S1 Data Excel spreadsheet containing the underlying numerical data for related figures. (XLSX)

S1 Text Chinese informed consent forms and corresponding English translation. (PDF)

## Abbreviations

cAMP: cyclic adenosine monophosphate
Cdx2: caudal type homeobox 2
CT: cytotrophoblast
DEG: differentially expressed gene
EM: endometrial
EPI: epiblast
EVT: extravillous trophoblast
GO: gene ontology
HERV: human endogenous retrovirus
ICM: inner cell mass
kME: intramodular gene connectivity
mESC: mouse embryonic stem cell
MOI: multiplicity of infection
NONOG: Nanog homeobox
PCA: principle component analysis
PE: primitive endoderm
POU5F1: POU class 5 homeobox 1
RNA-seq: RNA sequencing
RPM: read count per million
SCBAV: single-cell bifurcation analysis using variance of gene expression
scRNA-seq: single-cell RNA sequencing
SCUBA: single-cell clustering using bifurcation analysis
shRNA: short hairpin RNA
SNN: shared nearest neighbor
SOX2: SRY-box transcription factor 2
ST: syncytiotrophoblast
TBX3: T-box transcription factor 3
TE: trophectoderm
TOM: topological overlap matrix
UMS: ulnar-mammary syndrome
WGCNA: weighted gene co-expression network analysis
