# Supplementary figures and images for: Single-cell RNA sequencing reveals regulatory mechanism for trophoblast cell-fate divergence in human peri-implantation conceptuses
Source: PLoS Biol. 2019 Oct 9;17(10):e3000187. doi: 10.1371/journal.pbio.3000187 (PMC6802852; doi:10.1371/journal.pbio.3000187)

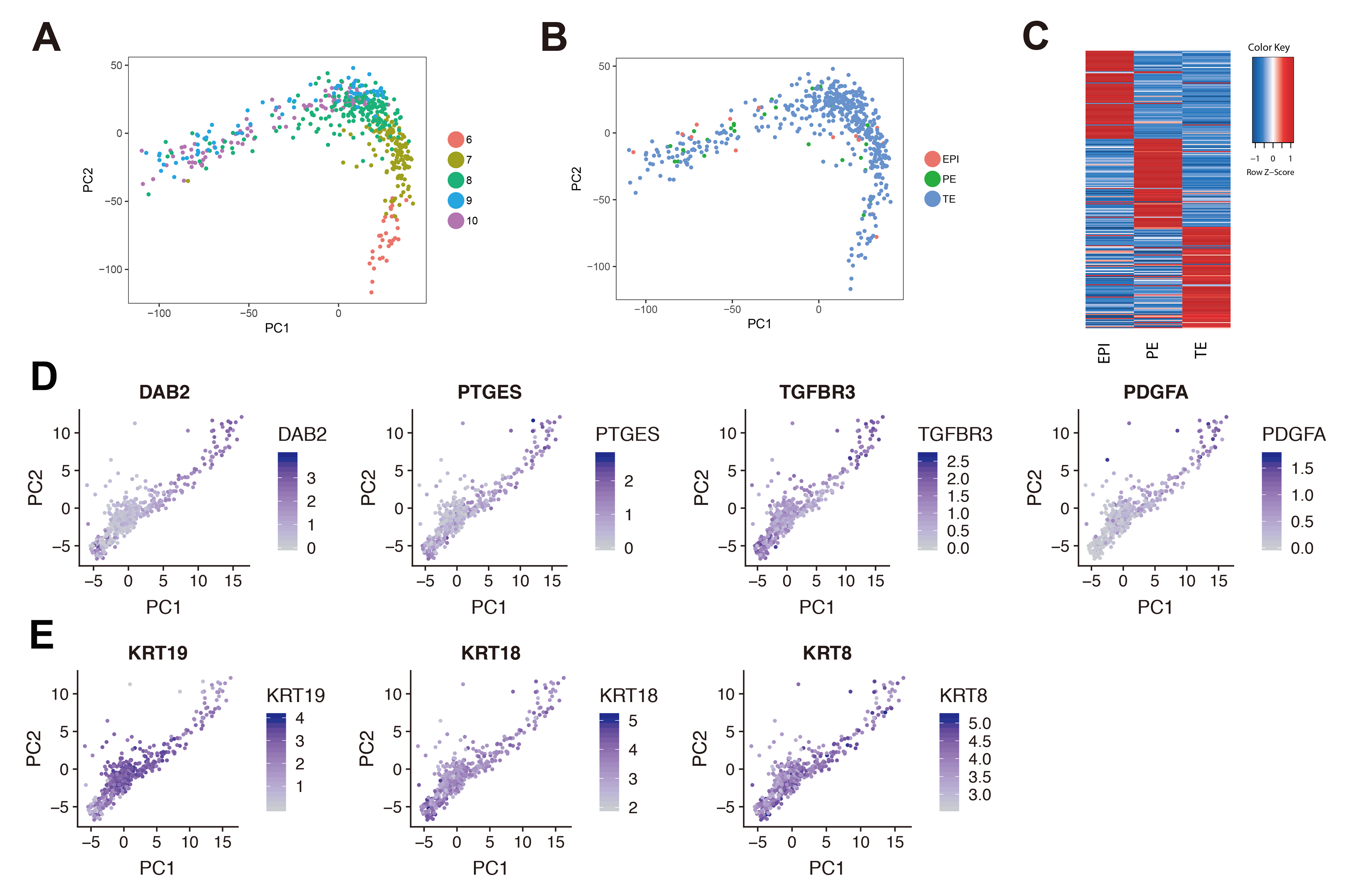

Supplement: S1 Fig — (A) PCA showing the unbiased clustering of day 6 through day 10 trophoblast cells, using all detected genes. (B) EPI-, PE- or TE-lineage cells were annotated based on Fig 2C. Cell identities were visualized on PCA related to Fig S1A. (C) Heat map showing the average expression of previously proposed markers among EPI, PE, and TE in 3 lineages. (D) Scatter plot showing the expression of previously identified TE-lineage markers. (E) Scatter plot showing the expression of highly expressed TE-lineage markers identified in this study. EPI, epiblast; PCA, principle component analysis; PE, primitive endoderm; TE, trophectoderm. (TIF) [file pbio.3000187.s001.tif]

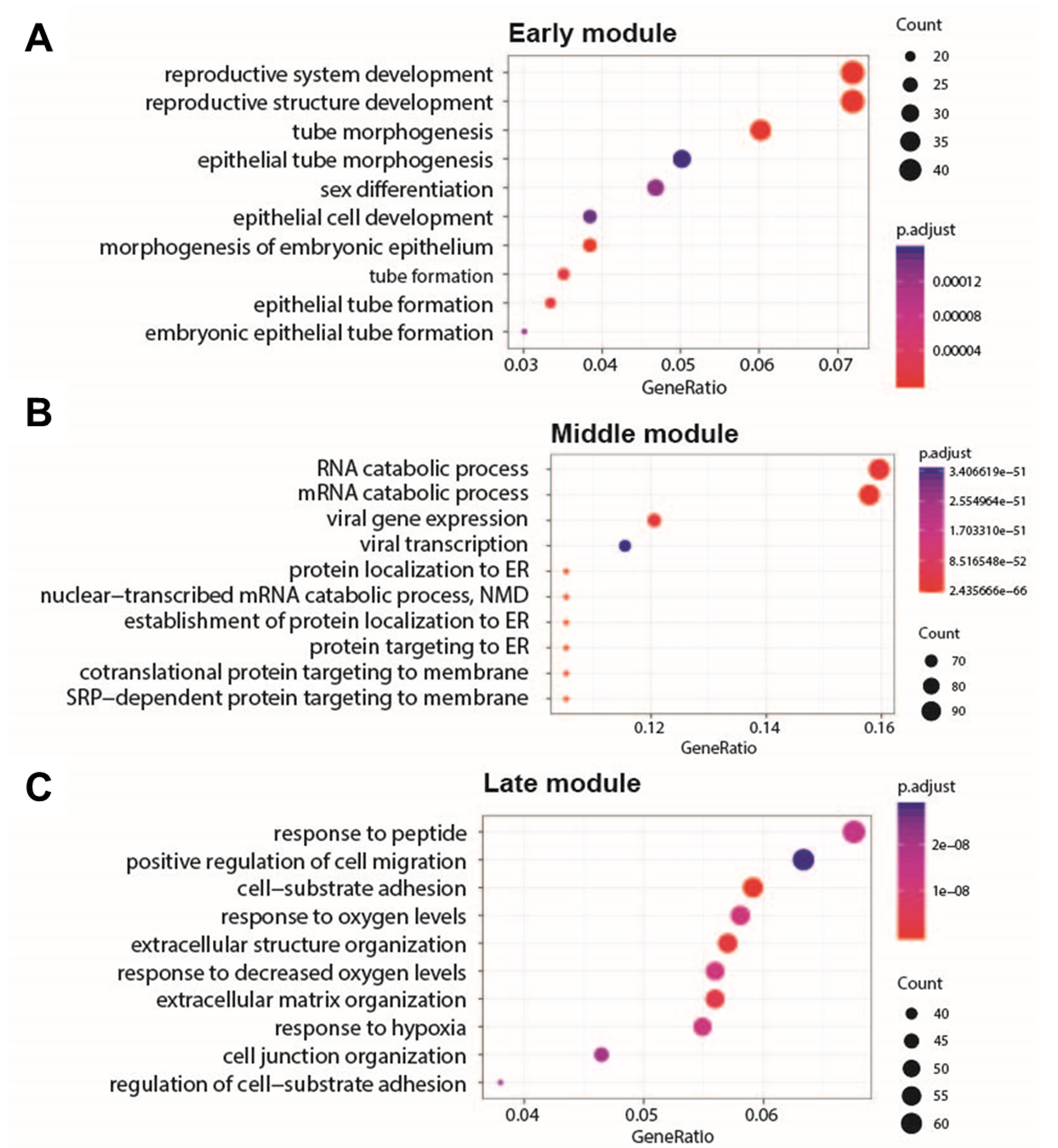

Supplement: S2 Fig — GO, gene ontology. (TIF) [file pbio.3000187.s002.tif]

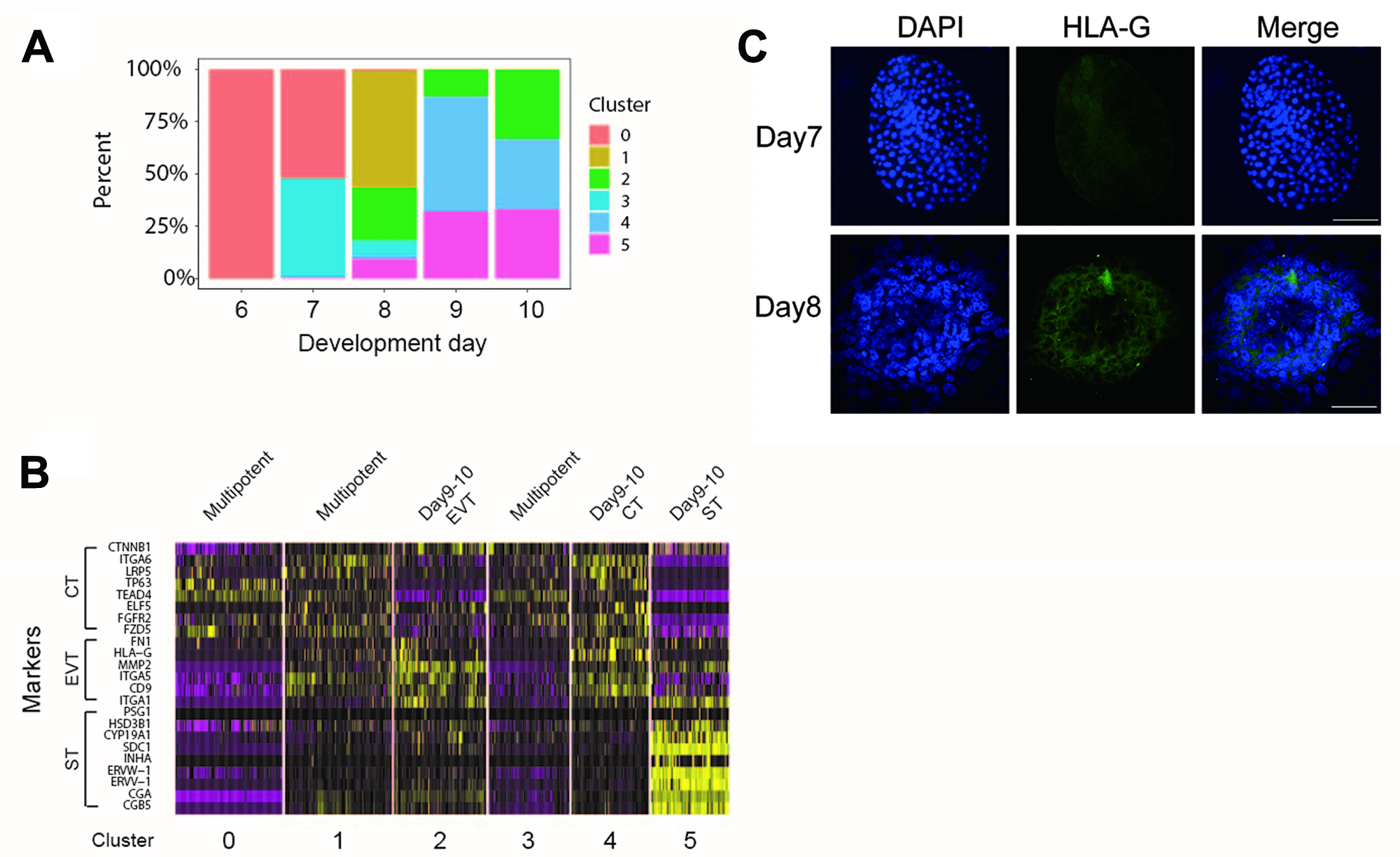

Supplement: S3 Fig — (A) Stacked bar plot showing the parentage of cells of 6 subpopulations at different development days. (B) Heat map showing the expression of previously identified CT, EVT, and ST markers in 6 trophoblast subpopulations. (C) Immunostaining of HLA-G in day 7 and day 8 conceptuses. (Scale bars = 100 μm.) CT, cytotrophoblast; EVT, extravillous trophoblast; HLA-G, human leukocyte antigen-G; RNA-seq, RNA sequencing; ST, syncytiotrophoblast. (TIF) [file pbio.3000187.s003.tif]

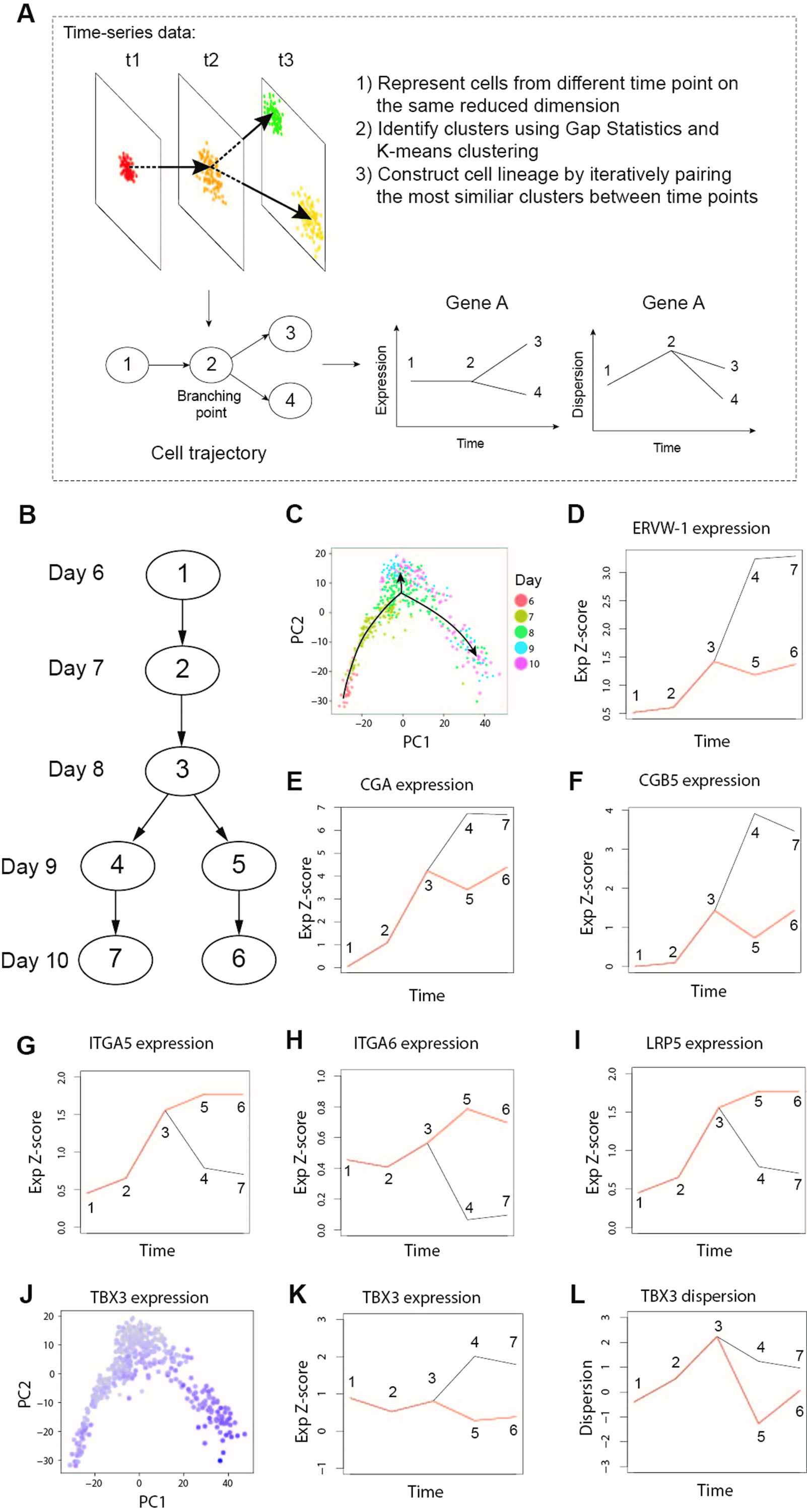

Supplement: S4 Fig — (A) Graphical abstract of SCBAV. (B) Cell trajectory reconstructed by SCBAV. (C) The bifurcation within the SCBAV cell trajectory recapitulated the cell-fate divergence of ST from CT and EVT. (D–F) Expression of ST specific genes within 2 lineage branches. (G–I) Expression of CT specific genes within 2 lineage branches. (J–L) TBX3 is variably expressed before bifurcation point and significantly up-regulated in ST compared with EVT and CT after bifurcation. CT, ytotrophoblast; EVT, extravillous trophoblast; SCBAV, single-cell bifurcation analysis using variance of gene expression; ST, syncytiotrophoblast; TBX3, T-box transcription factor 3. (TIF) [file pbio.3000187.s004.tif]

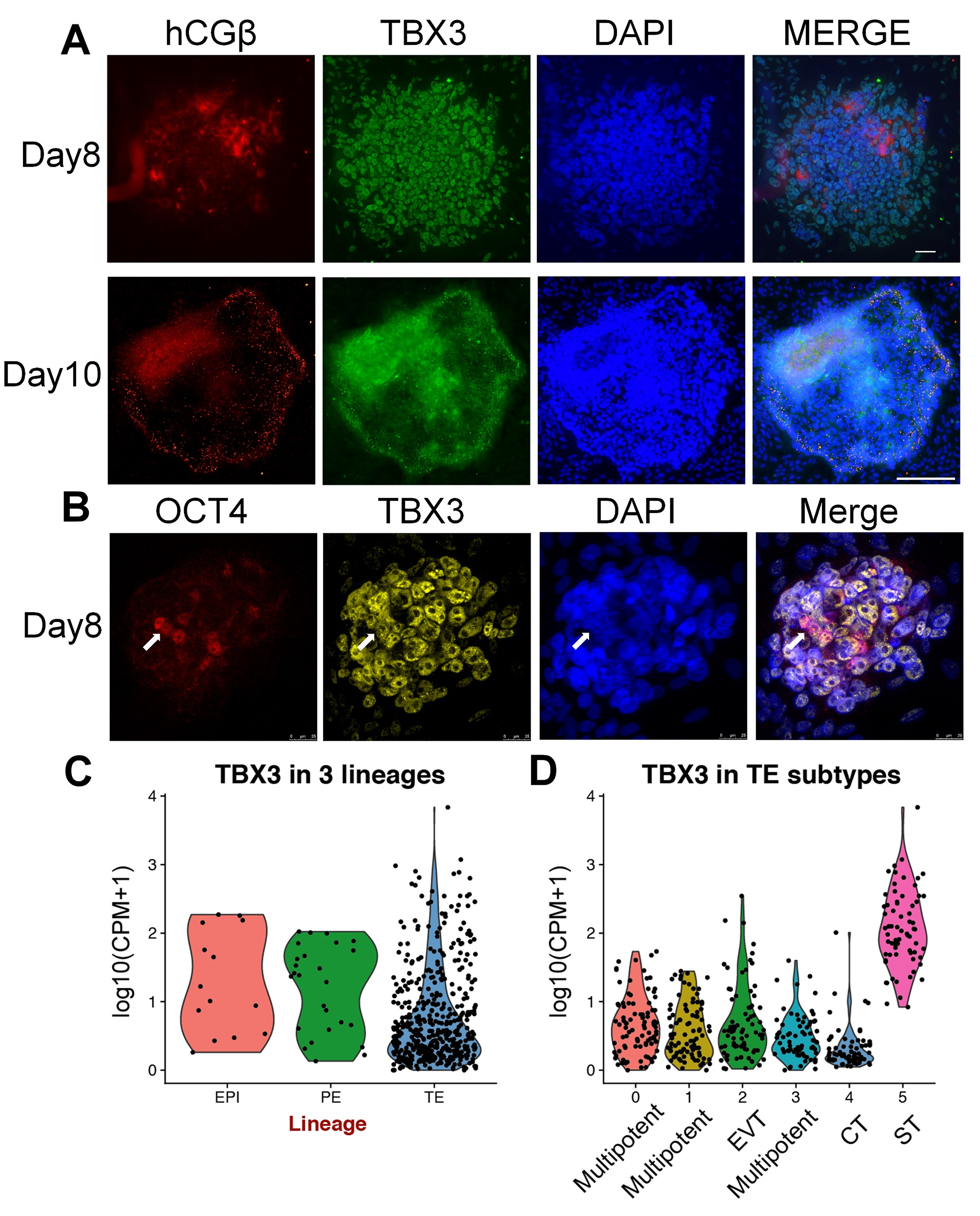

Supplement: S5 Fig — (A) Immunostaining of hCGβ and TBX3 in day 8 and day 10 conceptuses. Scale bars = 100 μm. (B) Immunostaining of OCT4 and TBX3 in day 8 and day 10 conceptuses. Scale bars = 50 μm. (C–D) Violin plot showing the expression of TBX3 in 3 conceptus lineages (C) and in different TE subtypes (D). OCT4, alias of POU class 5 homeobox 1 (POU5F1); TBX3, T-box transcription factor 3; TE, trophectoderm. (TIF) [file pbio.3000187.s005.tif]

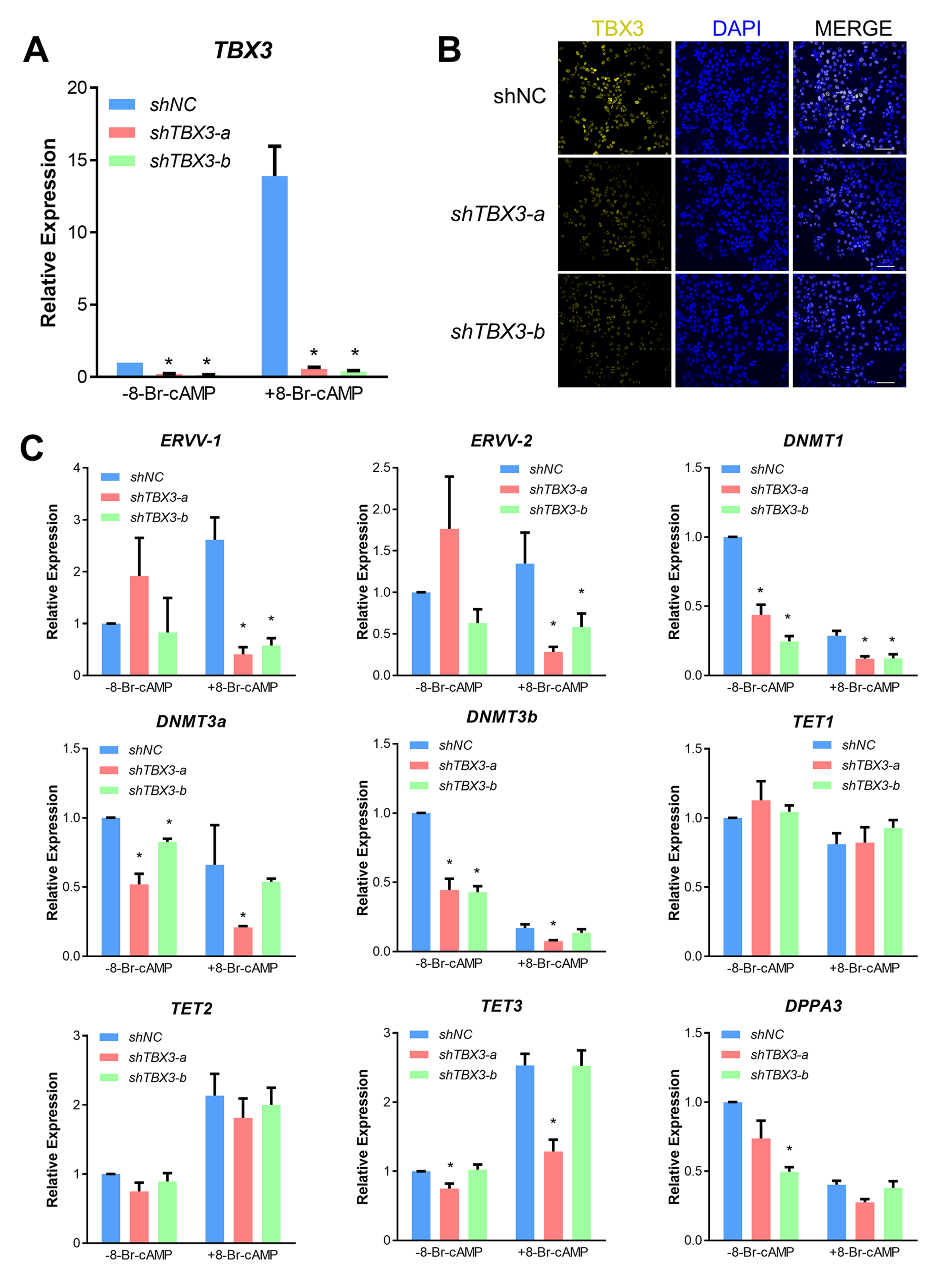

Supplement: S6 Fig — (A) and (C) qPCR for TBX3, ERVV-1, ERVV-2, DNMT1, DNMT3a, DNMT3B, TET1, TET2, TET3, and DPPA3 expression in JEG-3 cells expressing shNC, shTBX3-a, or shTBX3-b before or after 0.25 mM 8-Br-cAMP treatment for 48 h. *p < 0.05, n ≥ 3, mean ± SD. (B) Representative images of TBX3 expression in JEG-3 cells expressing shNC, shTBX3-a, or shTBX3-b cultured under 0.25 mM 8-Br-cAMP for 48 h. Underlying data for all panels included in this figure can be found in S1 Data. (Scale bars = 100 μm.) cAMP, cyclic adenosine monophosphate; qPCR, quantitative PCR; shNC, short hairpin negative control RNA; TBX3, T-box transcription factor 3. (TIF) [file pbio.3000187.s006.tif]

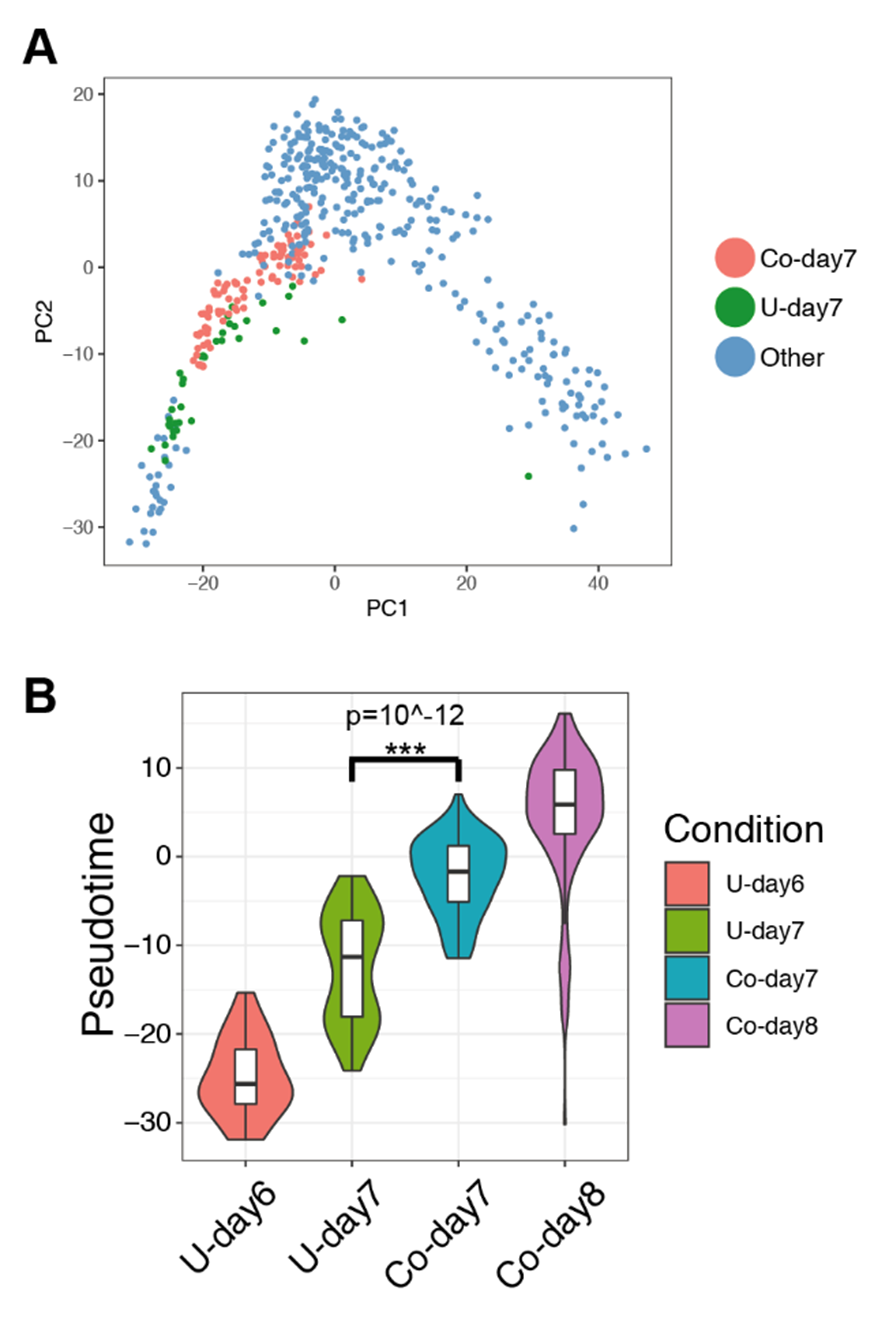

Supplement: S7 Fig — (A) PCA showing the 6 subpopulations within all trophoblasts. (B) Violin plot showing the pseudotime distribution of trophoblast population under different culture condition across different development days. The pseudotime of each cell is inferred using principal curve analysis. PCA, principle component analysis. (TIF) [file pbio.3000187.s007.tif]

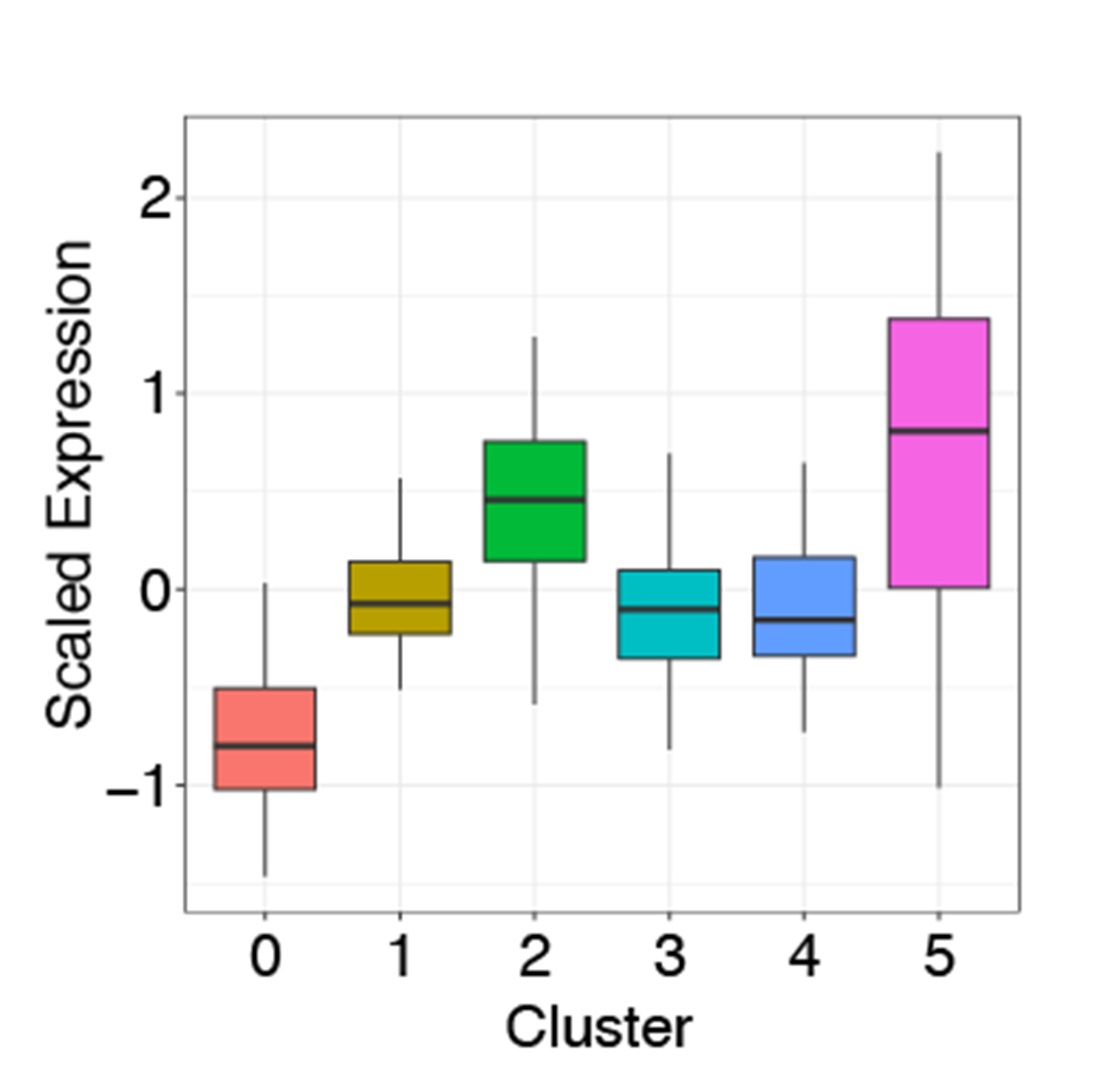

Supplement: S8 Fig — The average expression of polar genes are significantly higher in Cluster 2 (EVT day 9–10, p < 2.2 × 10^−16^) and Cluster 5 (ST day 9–10, p = 1.64 × 10^−14^) compared with other clusters (CT and multipotent trophoblasts). CT, cytotrophoblast; EVT, extravillous trophoblast; ST, syncytiotrophoblast; TE, trophectoderm. (TIF) [file pbio.3000187.s008.tif]

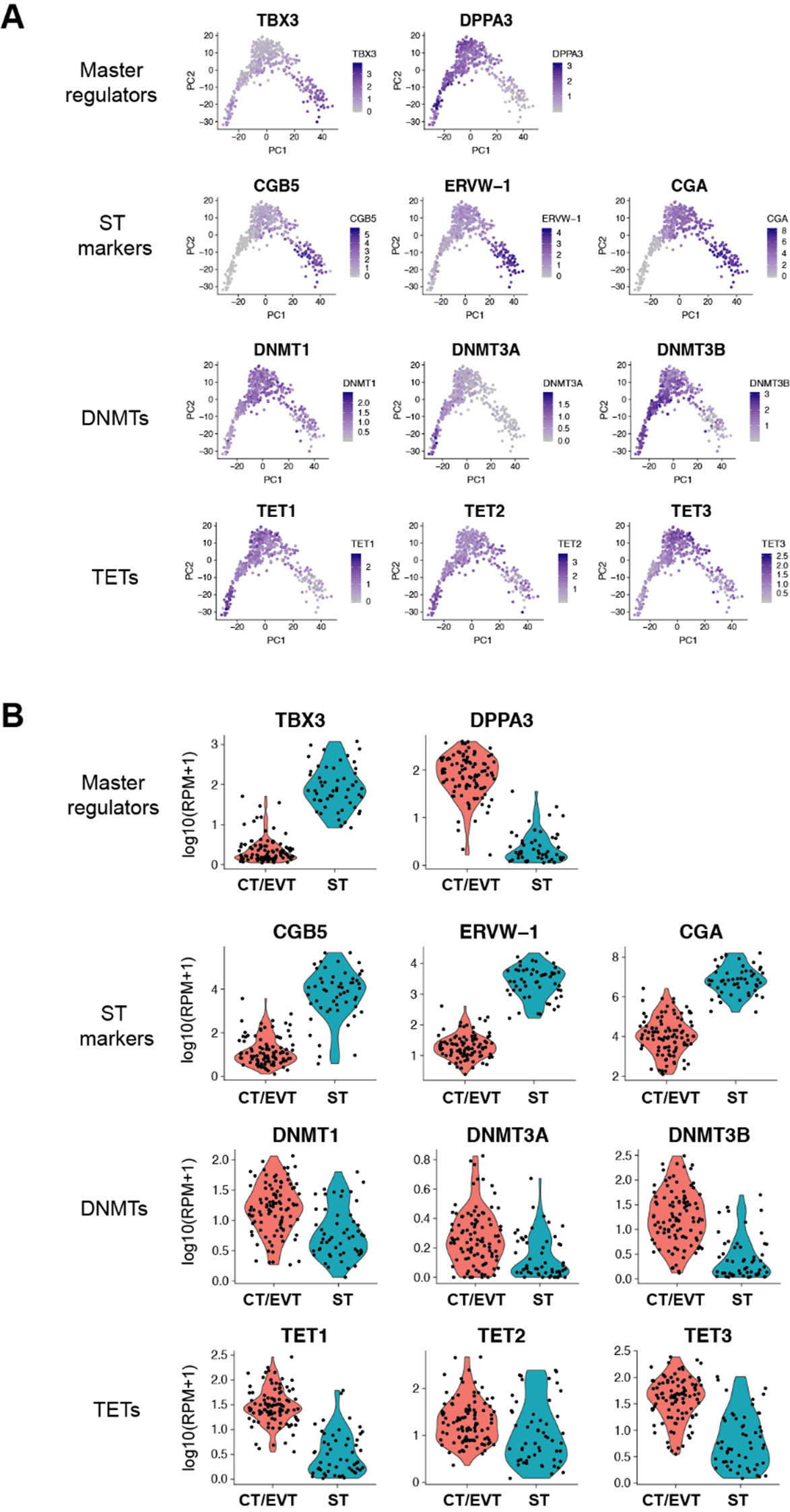

Supplement: S9 Fig — (A–B). Scatter plot and violin plot showing the expression of upstream regulators, ST marker genes, DNA methyltransferases, and TET methylcytosine dioxygenases. ST, syncytiotrophoblast; TET, ten-eleven translocation. (TIF) [file pbio.3000187.s009.tif]

**Table S1**: Summary of TE, EPI and PE cells across development days.

**
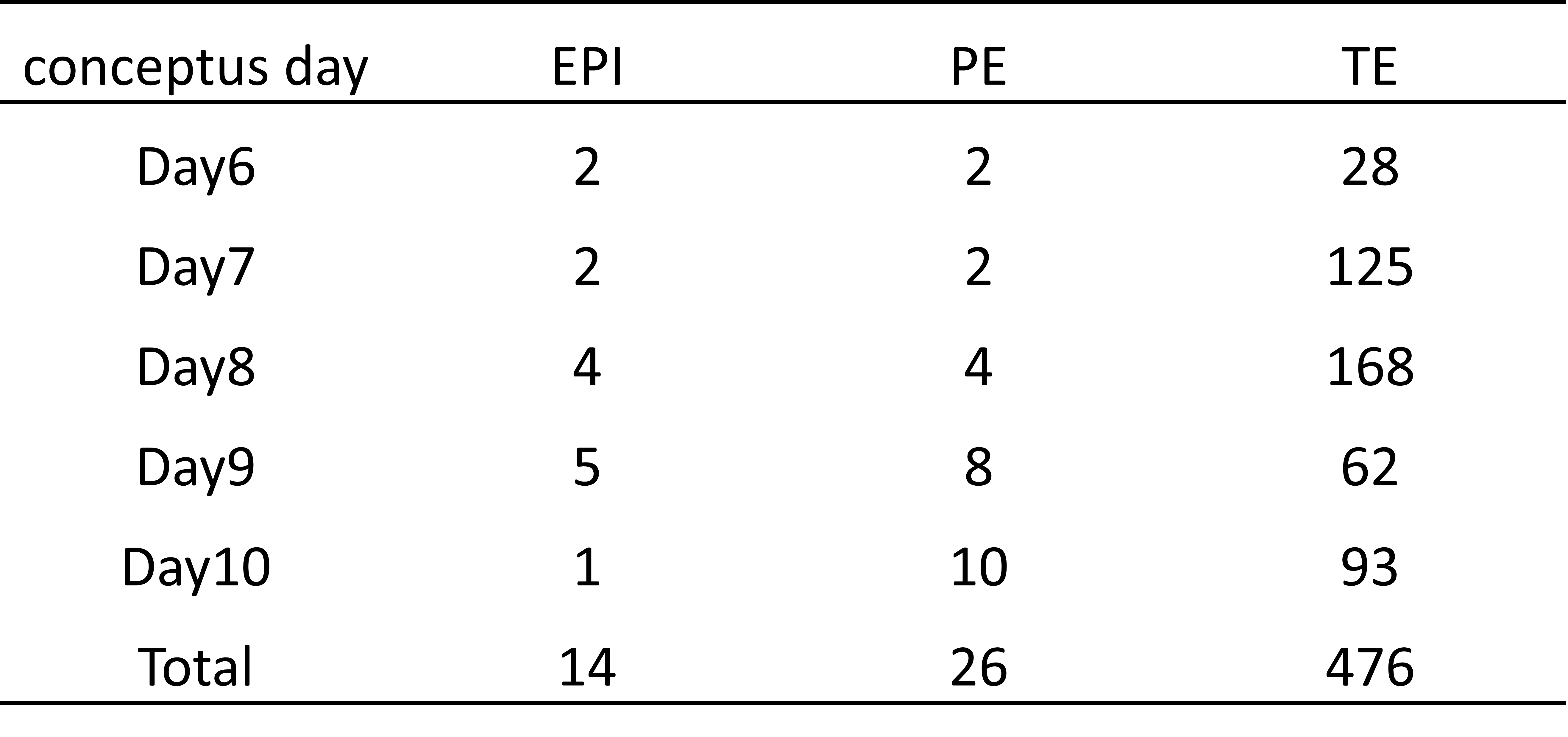
**

Supplement: S1 Table — EPI, epiblast; PE, primitive endoderm; TE, trophectoderm. (DOCX) [file pbio.3000187.s010.docx]

**Table S2:** Statistical analysis of *DAB2*, *PTGES*, *TGFBR3* and *PDGFA* expression between day6 and day7.

**
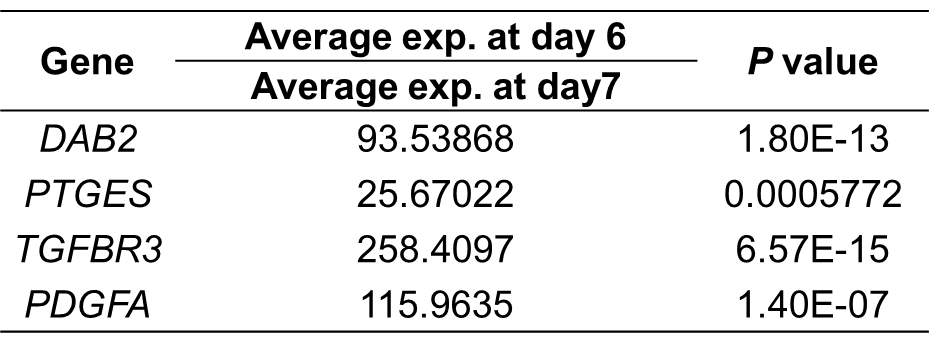
**

Supplement: S2 Table — (DOCX) [file pbio.3000187.s011.docx]

**Table S3**: Summary of six trophoblast clusters across development days.


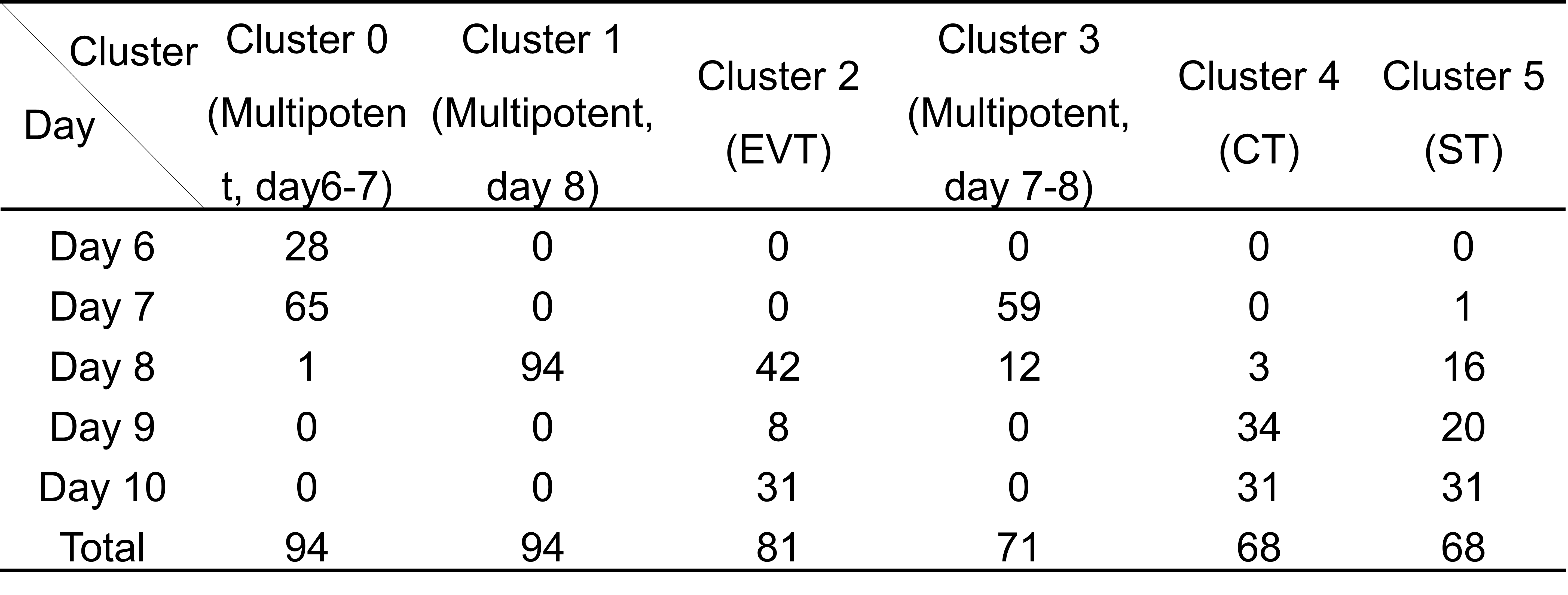

Supplement: S3 Table — (DOCX) [file pbio.3000187.s012.docx]
