# Supplementary material for: Single-cell RNA sequencing reveals regulatory mechanism for trophoblast cell-fate divergence in human peri-implantation conceptuses
Source: PLoS Biol. 2019 Oct 9;17(10):e3000187. doi: 10.1371/journal.pbio.3000187 (PMC6802852; doi:10.1371/journal.pbio.3000187)
Supplement: S7 Table — qRT-PCR, quantitative real-time PCR. (DOCX) [file pbio.3000187.s016.docx]

**Table S7**: Primers used for qRT-PCR

| Gene | Forward Primer | Reverse Primer |
| --- | --- | --- |
| *ERVV-1* | CCTCTGTTCCAGAAGGGAACT | TGGGGAATAGCTCCTACCTTG |
| *ERVV-2* | GAAACGCAGCCCACTGATAG | GGATGGGTTATTAGAGAAGTGCTG |
| *ERVW-1* | CTTCCTCTCATTCTTAGTGCCC | CCAATGCCAGTACCTAGTGC |
| *TBX3* | AAAAATAGACAACAACCCTTTTGC | ACTGCAGGGTGAGCTGTTTT |
| *DPPA3* | TCGAAGATGAGTGGCTTTACAG | CTTAACTCCCTTAGGCTCCTTG |
| *CGA* | CATTGTCGGTGTTTCTGCATG | TGGGTTTTCCTGTAGCGTG |
| *CGB* | CTGGCTGTGGAGAAGGAG | ACATCGCGGTAGTTGCAC |
| *DNMT1* | CCAGAGAACGAGTTGCTAGAC | CAGTTTCTGTTTGGGTGTTGG |
| *DNMT3a* | TCTCTTTGATGGAATCGCTACAG | GTACATGATCTTCCCCTGGTG |
| *DNMT3b* | CCCATTCGAGTCCTGTCATTG | TTGATATTCCCCTCGTGCTTC |
| *TET1* | TTCGTCACTGCCAACCTTAG | ATGCCTCTTTCACTGGGTG |
| *TET2* | TTTGTTAGAAAGGAGACCCGAC | CAGGAGCAAAGGCAAGTAAAC |
| *TET3* | CGCCTCTATCCGGGAACT | CTTCCCCGTGTAGATGACCT |
| *GAPDH* | ACATCGCTCAGACACCATG | TGTAGTTGAGGTCAATGAAGGG |
