# Supplementary material for: Single-cell RNA sequencing reveals regulatory mechanism for trophoblast cell-fate divergence in human peri-implantation conceptuses
Source: PLoS Biol. 2019 Oct 9;17(10):e3000187. doi: 10.1371/journal.pbio.3000187 (PMC6802852; doi:10.1371/journal.pbio.3000187)
Supplement: S1 Text — (PDF) [file pbio.3000187.s018.pdf]

知情同意书

研究机构：同济大学附属同济医院

主要研究者：同济大学附属同济医院纪亚忠教授、薛志刚教授

您将被邀请参加一项胚胎发育过程的分子机制研究。本知情同意书提供给您一些信息以帮助您决定是否参加此项临床研究。请您仔细阅读，如有任何疑问请向研究人员提出。

本次研究已通过本研究机构伦理审查委员会审查。如果您有与受试者自身权益相关的问题，**可与同济大学附属同济医院/上海市同济医院伦理委员会联系，联系电话：021-66111243，电子邮件：tongjilunli2012@163.com**。

研究目的：

阐明体外与子宫内膜细胞培养围植入期胚胎发育的分子机制，探讨植入失败的原因。

研究背景和意义：

辅助生殖过程中约50%胚胎在移植后着床失败，且缺乏人类囊胚植入的分子机制研究。本项目将利用单细胞测序技术和人体外囊胚植入模型解析胚胎植入基因表达规律，解析相应的分子调控网络。

入选标准和排除标准：

1. 年龄：20-40岁；2. 染色体正常；3. 已获得至少一个健康后代。

研究过程：

收集检查后剩余子宫内膜组织，消化成分散的细胞。收集发育到囊胚阶段的胚胎，与子宫内膜细胞进行共培养。收集围植入期胚胎样本，经消化后获得单细胞，裂解并进行相应处理，进行转录组等研究，进而分析关键基因在围植入期胚胎发育过程中的作用。

如果您同意参与这项研究，我们将对每位受试者进行编号，建立研究档案。在研究过程中我们需要采集一些您的基本信息。您的样品仅用于该研究。

风险与不适：

对于您来说，所有的信息将是保密的。因为所捐赠的内膜组织是一些临床检查剩余的样本，胚胎是因成功获得健康后代后剩余或废弃的胚胎，所以本研究不会对您造成额外风险和不适。

受益：

研究结束后我们都将对这些生物材料处理失活而不会损害捐赠者的潜在利益。所有捐赠都是自愿、无偿的，且捐赠者将不再拥有对所捐赠生物材料的权益，研究成果将归研究方所有。

我们保证所有捐赠的胚胎、内膜组织不会用于任何商业和营利性行为。我们的研究遵守国家相关伦理法规。同济大学附属同济医院伦理委员会已经审议通过我们所述的研究，认为我们的研究合乎医学伦理，并批准了研究内容。

研究相关费用：

参加本研究项目不会增加您额外支出，本研究的所有实验费用等由研究经费承担。

发生伤害的医疗及赔偿：

本研究是采用剩余或则废弃的内膜组织和胚胎，对您不会造成任何损害。

即使您已签署这份知情同意书，您仍然保留您所有的法定权益。

如果您因参与这项研究而受到伤害，您可以获得免费治疗和／或相应的补偿。

作为研究受试者，您有以下职责：

提供有关自身病史和当前身体状况的真实情况；告诉研究医生自己在本次研究期间所出现的任何不适；不得服用受限制的药物、食物等；告诉研究医生自己在最近是否曾参与其他研究，或目前正参与其他研究。

隐私和保密：

如果您决定参加本项研究，我们将在法律允许的范围内，尽一切努力保护您的个人隐私。研究医师及其他研究人员将使用您的医疗信息进行研究。这些信息可能包括您的姓名、地址、病史及在您来访时得到的信息。您身份的信息将不会透露给研究小组以外的成员，除非获得您的许可。所有的研究成员和研究相关方都被要求对您的身份保密。您的档案将保存在有锁的档案柜中，仅供研究人员查阅。为确保研究按照规定进行，必要时，政府管理部门或伦理审查

委员会的成员按规定可以在研究单位查阅您的个人资料。任何有关本项研究结果的公开报告不会披露您任何的个人信息。

研究期间，任何与您健康有关的有意义的新进展或新医疗信息，我们将及时联系您，如建议您进行检查来确定这些新信息等。任何可能影响您选择是否继续参加研究的所有新信息，我们也会及时告知您。

受试者权利：

是否参加研究完全取决于您的自愿。您可以拒绝参加此项研究，或在研究过程中的任何时间退出本研究，您的数据将不纳入研究结果。这都不会影响您和医生间的关系。您的任何医疗待遇与权益不会因此而受到影响。

如果您需要其它治疗，或者您没有遵守研究计划，或者发生了与研究相关的损伤或者有任何其它原因，研究医师可以终止您继续参与本项研究。

知情同意书签字页

我已经阅读本知情同意书，已就此项研究与医生讨论并提出问题。医生已经将研究的目的、研究过程、可能遭受的风险和受益向我作了详细的解释说明，对我的所有问题也给予了解答，我知晓参加本研究是自愿的。

我确认已有充足时间对此进行考虑，包括参加研究可能产生的风险。我可以随时向医生咨询更多的信息、可以随时退出本研究而不会受到歧视和报复、医疗待遇与受益不会因退出研究而受到影响。

我自愿参加本项研究。相关人员将有权使用捐赠的标本以用于人类早期胚胎发育机制的研究。

我将获得一份经过签名并注明日期的知情同意副本。

捐赠者签名：________________________

日期：_______年________月________日

我已准确地将这份文件告知受试者，他/她准确地阅读了这份知情同意书，并证明该受试者有机会提出问题。我证明他/她是自愿同意的。

研究者签名：________________________

日期：______ 年________月________日

Informed Consent Form (English Translation)

Research Institution: Tongji Hospital of Tongji University

Project Leaders: Prof. Yazhong Ji, and Prof. Zhigang Xue in Tongji Hospital of Tongji University

You are invited to participate in a research on the molecular mechanism of the embryonic development. This informed consent will provide you the information to help you decide to involve in this study or not. Please read this informed consent very carefully, and you can ask any question at any time if you are in any doubt.

This study has been approved by the Institutional Review Board (IRB) of Tongji Hospital in Tongji University. If you have any question related to the participants' right, please contact the Institutional Review Board (IRB) of Tongji Hospital in Tongji University (Shanghai Tongji Hospital), Tel: 021-66111243, Email: tongjilunli2012@163.com.

Research purpose:

The purpose of this study is to elucidate the mechanism of peri-implantation embryonic development in vitro when cocultured with endometrial cells and to explore the reason of implantation failure.

Research background

In assisted reproductive progress, about 50% of embryos fail to implant after transferring to uterus and the mechanism of blastocyst implantation remains unclear in human. This study will utilize single cell sequencing technology and in vitro human blastocyst implantation model to dissect the gene expression pattern of embryo implantation and clarify the related molecular network.

Inclusion criteria and exclusion criteria:

1. Age: 20-40; 2. Normal chromosome; 3. Couples with at least one healthy offspring.

Research progress:

We will collect leftover endometrium tissues after clinical examination and dissociate them to dispersed cells. We will collect embryos to culture them into blastocyst stage and then co-culture them with endometrial cells in vitro. We will get

the in vitro peri-implantation embryo samples and dissociate them into single cells, then lyse and carry out the study, such as transcriptome analysis. Then we will analyze the function of key genes in peri-implantation embryonic development.

If you agree to participate in this study, you will be numbered and a research archive will be established. We need to collect some basic information about you during the research process. Your sample is for this study only.

Risk and the possible harm:

All the information of you will be confidential. Because the donated endometrium tissues are from the samples left by the clinical examine and leftover embryos from the couples who have successfully had the healthy child, this research will not cause additional risk to you.

Benefit:

We will inactivate these biomaterials at the end of our research to not harm the potential benefit of participants. All the donations are voluntary and the donators will no longer have the right to the donated biomaterials. The research results will belong to the researchers.

We guarantee that all donated embryos and endometrial tissues will not be used for any commercial or profitable activities. Our research complies with relevant national ethics and regulations. The IRB of Tongji Hospital in Tongji University has discussed and adopted our research. The IRB believed our research is in line with the medical ethics and approved the content of the research.

Research related expense:

The researcher will take over all the expense involved in the study. You do not need to pay any extra cost.

Risk management and compensation for injuries:

Because the donated endometrium tissues are from the samples left by the clinical examine and leftover embryos from the couples who have successfully had the healthy child, this research will not cause additional health risk.

Even if you have signed this informed consent, you will still retain all your legal rights and interests.

If you get hurt caused by this study, you will get free cure and/or related compensation.

The duties of participants:

You should provide the information about your medical history and current physical condition; tell the research doctor if you have any discomfort during this study, do not take restricted drugs, food, etc; tell the research doctor whether you have participated in other research recently or is currently participating in other research.

Privacy and confidentiality:

If you decide to participate in this research, we will make every effort to protect your personal privacy within the scope of the law. Doctors and other researchers in this study will use your medical information for the research purpose. The information may include your name, address, medical history, and other information when you come to our hospital. Without your permission, any of your information would not be disclosed to any people outside the research group. All the relevant researchers are required to keep your identity confidential. Your file will be kept in the locked filling cabinet. Only relevant researchers could read your information. When necessary, people from the regulation department of the government, university or the Ethics committee member may also check your information. Any of your personal information would not be disclosed when any relevant research work get published in the future.

During this research, we will contact you in time for any meaningful new developments or medical information related to your health and recommend that you check these new information. We will also inform you of any new information that may affect your choice of whether or not to continue your research.

Rights of participants:

Whether you participate in the research or not depends entirely on your willingness. You may refuse to participate in the study or withdraw from the study at any time during the course of this study. Your data will not be included in the results of the study. This will not affect your relationship with your doctor. Your medical treatment and rights will not be affected.

If you need other treatment, or if you do not follow the research plan, or if you have a research-related injury or for any other reason, the researcher may terminate your participation in the study.

Informed consent signature page

I have read this Informed Consent Form, discussed this study with my doctor and asked questions. The doctor has explained to me in detail the purpose, process, risks and benefits of the study and also answered all my questions. I know I am voluntary to participate in the study.

I confirm that there is sufficient time to consider this, including the risks that may arise from participating in the study. I can consult my doctor for more information at any time, and withdraw from the study at any time without discrimination or retaliation. Medical treatment and benefits will not be affected by withdrawal from the study.

I volunteered to participate in this research. Relevant researchers will have the right to use donated samples for the study of mechanism during human early embryonic development.

I will get a signed and dated copy of informed consent.

Donor Signature:

Date:

I have accurately informed the participant of this document. He/she has read the informed consent accurately and has demonstrated that the participant has the opportunity to ask questions. I certify that he/she consented voluntarily.

Witness Signature:

Date:
